# Biogeography of the Iranian snakes

**DOI:** 10.1371/journal.pone.0309120

**Published:** 2024-10-16

**Authors:** Naeim Moradi, Ulrich Joger, Soheila Shafiei Bafti, Ali Sharifi, Mohammad Ebrahim SehhatiSabet

**Affiliations:** 1 Iranian Plateau Herpetology Research Group (IPHRG), Faculty of Sciences, Razi University, Kermanshah, Iran; 2 Zoological Institute, Technical University of Braunschweig, Braunschweig, Germany; 3 Department of Biology, Faculty of Sciences, Shahid Bahonar University of Kerman, Kerman, Iran; 4 Monetary and Banking Research Institute, Tehran, Iran; 5 Department of the Environment of Iran, Provincial Office of Kerman, Kerman, Iran; University of Basrah, IRAQ

## Abstract

The events of the Cenozoic era such as mountain formation caused Iran to become one of the most amazing biodiversity hotspots in the world today. This pioneering study on Iranian snake biogeography integrates historical and ecological analyses. A phylogeographic review traces speciation and dispersal, while cluster analysis with a new snake checklist assesses faunistic similarities within Iran and its surroundings. Jaccard and Sorenson indices generate similarity dendrograms, Indicator Species Analysis pinpoints regional key species, and Endemism index calculates regional endemism rates, enriching our knowledge of Iran’s species diversity. Phylogeographic analyses identify four biogeographical corridors for snake ingress into Iran: the Arabian region through southwestern Iran, the Western Asian mountainous transition zone via northwestern Iran, the Turanian region into northeastern Iran, and the Indus River Valley into southeastern and eastern Iran. Dendrogram analysis divides snake fauna into three groups. The first group associates western Zagros and Khuzestan fauna with the Sahara and Arabian regions. The second group links Kopet Dagh and Turkmen Steppe fauna with the Turanian region, and Central Plateau and Baluchistan fauna with the Iranian region. The third group connects northwest highlands, Alborz and Zagros mountains, and Caspian Sea coasts with the Western Asian Mountain transition zone. The study validates broad biogeographic patterns via ecoregional associations and indicator species analysis, providing finer resolution. Species like *Platyceps najadum* in Caspian Hyrcanian mixed forests exemplify ecoregional alignment, while Zagros and Alborz mountains exhibit unique faunal indicators, indicating species-level divergence. Shared indicators among widespread ecoregions reflect habitat continuity; exclusive indicators emphasize regional distinctiveness. Despite endemic species prevalence, they seldom act as significant indicators due to various factors. Our research confirms the Zagros Mountains, Khuzestan Plain, Alborz Mountains, and Persian Gulf coasts as snake diversity hotspots, marked by higher species richness compared to other Iranian regions.

## Introduction

The Iranian Plateau began forming during the Late Cretaceous period (100–66 Mya) due to the initial convergence and collision of the Arabian and Eurasian tectonic plates [1]. This collision resulted in the closure of the Neo-Tethys Ocean and created the foundation for the plateau. In the early Cenozoic era, specifically the Paleocene and Eocene epochs (66–34 Mya), the Arabian plate continued to push northward, leading to significant crustal shortening and thickening across central Iran [2]. This created a high topography and the initial uplift of the Iranian Plateau. During the Oligocene epoch (34–23 Mya), Arabia-Eurasia convergence rates increased, triggering major deformation and faulting events, especially in the Zagros Mountains along the southwestern plateau margin [3]. This caused further uplift and shaping of the plateau’s relief. The Arabia-Eurasia collision zone shifted northward in the Miocene epoch (23–5 Mya), leading to immense uplift, faulting, and folding across the plateau. This also led to the formation of the Alborz Mountains along the northern plateau boundary [4]. By the end of the Pliocene epoch (2.5 Mya), the geologic forces from the ongoing convergence had raised the Iranian Plateau to largely its present-day elevation and extent [2]. However, active tectonics continue to impact plateau landforms in the Quaternary period (2.5 Mya—present).

Phylogeography combines data on geographic distributions, genetic relationships, and evolutionary timescales to reveal how historical events have shaped genetic diversity and lineage distributions. Factors like past climate change, mountain formation, sea level fluctuations, and habitat fragmentation can drive vicariance, dispersal, range shifts, and population divergence. Molecular dating methods provide temporal context. By integrating phylogeographic, fossil, paleoclimatic, and geological evidence, the complex biogeographic histories of snake lineages are coming into focus. However, many regions remain understudied, and cryptic diversity likely awaits discovery [5, 6].

The distribution of biodiversity is often uneven and influenced by both historical and contemporary factors [7]. This is especially evident in global hotspots like the Irano-Anatolian region [8, 9]. While this area is recognized for its high endemism and biodiversity [10], it faces habitat degradation and is under-explored [11]. Reptiles are suitable for analyzing historical biogeography in the region due to their low dispersal abilities and narrow niches [12]. However, reptile ecology and distributions remain poorly documented in many areas [13]. Factors like past climate fluctuations and mountain formation have likely shaped present-day reptile richness gradients and endemism patterns [14, 15]. More surveys and habitat assessments are needed, particularly in Iran. Analyzing genetic diversity is also crucial but lacking [16]. Exploring how historical versus contemporary factors drive reptile diversity in the region can guide conservation priorities [17, 18]. Overall, the Irano-Anatolian hotspot exemplifies the complex interplay between plate tectonics, climate, and dispersal limitations in shaping biodiversity gradients [19, 20]. Targeted assessments of reptile distributions and genetics are needed to elucidate these dynamics further [21].

**Fig 1:**
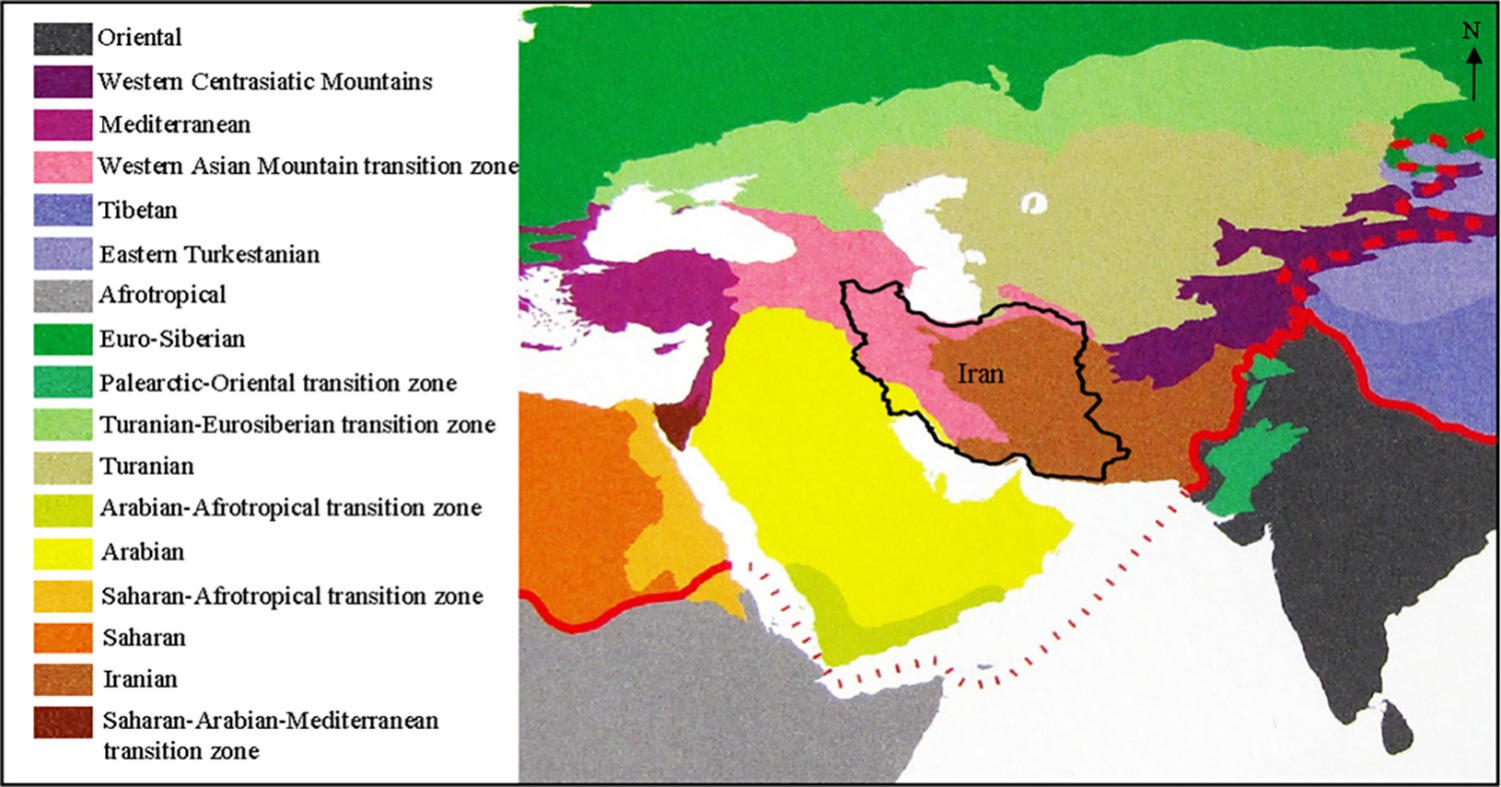
Biogeographic subdivision of Western Palearctic based on distribution of lizards [22]. Reprinted from [22] under a CC BY license, with permission from Edizioni Belvedere, original copyright 2008.

Sindaco *et al*. divided the Western Palearctic into seventeen subregions and transition zones based on lizards’ distribution [22]. Most of these areas (with the rejection of a unique Mediterranean subregion) are supported by the cluster analysis by Ficetola *et al*., based on the distribution of all reptile species [7]. According to this division, Iran contains parts of four of these regions (Fig 1). Two have a limited extent in Iran, including “Arabian” which extends to western and southwestern Iran, and “Turanian” which extends in a small portion of northern Iran. However, Iranian species are widespread mostly in the Central Plateau and on the inner slopes of the Zagros and Alborz Mountains, which is nominated as “Iranian”. The Zagros and Alborz Mountains belong to the “Western Asian Mountains Transition Zone”, a broad and morphologically complex area of western Asia, with a high rate of endemism, where species of different biogeographical areas coexist.

On the other hand, Anderson divided the Iranian lizard fauna into five elements [23]: “Iranian”, “Aralo-Caspian” (= Turanian), “Mediterranean”, “Saharo-Sindian”, and “Oriental”. Later on, to discuss the biogeography of Iranian lizards, he recognized thirteen biogeographic regions [24].

Considering the biogeography of amphibian and reptiles distributed in Iran, species richness was investigated by Iranian herpetologists in recent years as follow: reptile species of Iran [25, 26]; some amphibian and reptile species in the Zagros Mountain [27]; lizard species of Iran [20, 28]; and two families of lizards in Iran [29].

The primary objectives of this study are to compile an up-to-date checklist of species and subspecies, conduct a comprehensive phylogeographic review of all taxa based on existing studies, and highlight the patterns of taxa penetration into Iran, focusing on dispersal events. The study also aims to investigate the zoogeographic links between Iran’s physiographic regions and adjacent areas, identify indicator species in each area based on their respective ecoregions to discern species preferences, and examine species richness and endemicity rate to pinpoint areas of high biodiversity.

## Materials and methods

### Study area

The Iranian Plateau is a biogeographically diverse region situated at the intersection of several ecozones. It spans approximately 2 million km^2^ across Iran, Afghanistan, Pakistan and southern Turkmenistan [24]. This arid highland plateau is delineated by mountain ranges including the Alborz Mountains in the north and the Zagros Mountains along the western and southern edges [24]. These mountains create sharp environmental gradients, transitioning from mesic coastal lowlands to increasingly arid and continental conditions towards the plateau interior [30]. The plateau can be divided into several distinct biogeographic zones. The Hyrcanian zone occurs along the humid Caspian coast and contains relicts of Tertiary subtropical broadleaf forests. The Irano-Turanian zone covers much of the central and eastern plateau, consisting of arid steppes and deserts. The Saharo-Arabian zone occurs in the lowland southwest. Along the southern coast is the Sudanian zone with tropical savanna [31]. These zones have high levels of biodiversity and endemism.

**Fig 2:**
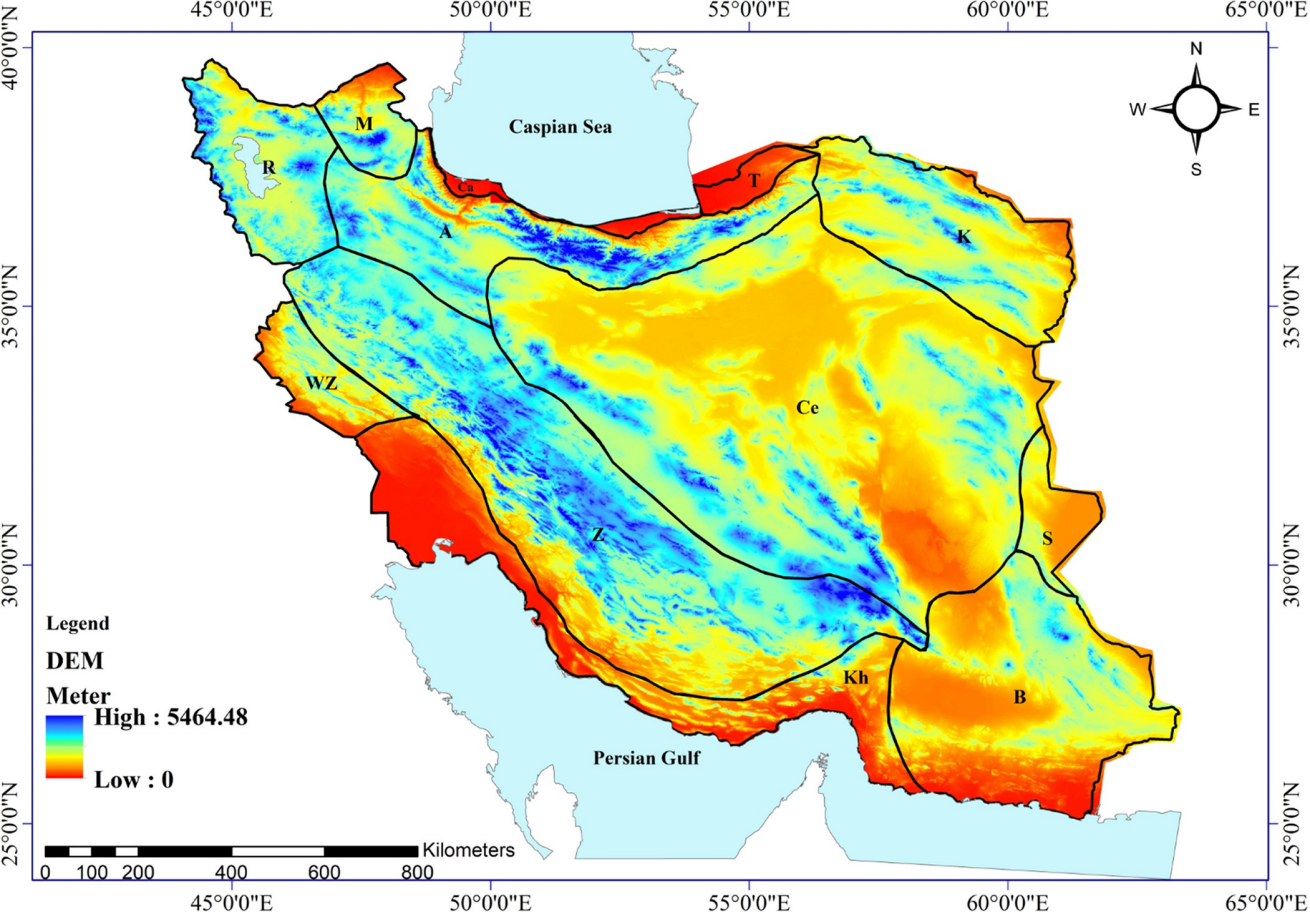
Physiographic subdivision of Iran based on the distribution of lizards [24]. Ce—Central Plateau; Ca—Caspian; A—Alborz Mountains; M—Moghan Steppe; R—Reza’iyeh (Urmia) Basin; Z—Zagros Mountains; WZ—Western Foothills of the Zagros Mountains; Kh—Khuzestan Plain and the Persian Gulf Coast; T—Turkmen Steppe; K—Kopet-Dagh; S—Sistan Basin; B—Iranian Baluchistan and the Makran Coast; I—Islands of the Persian Gulf. Digital elevation model (DEM) layer prepared from: https://dwtkns.com/srtm30m/.

**Fig 3:**
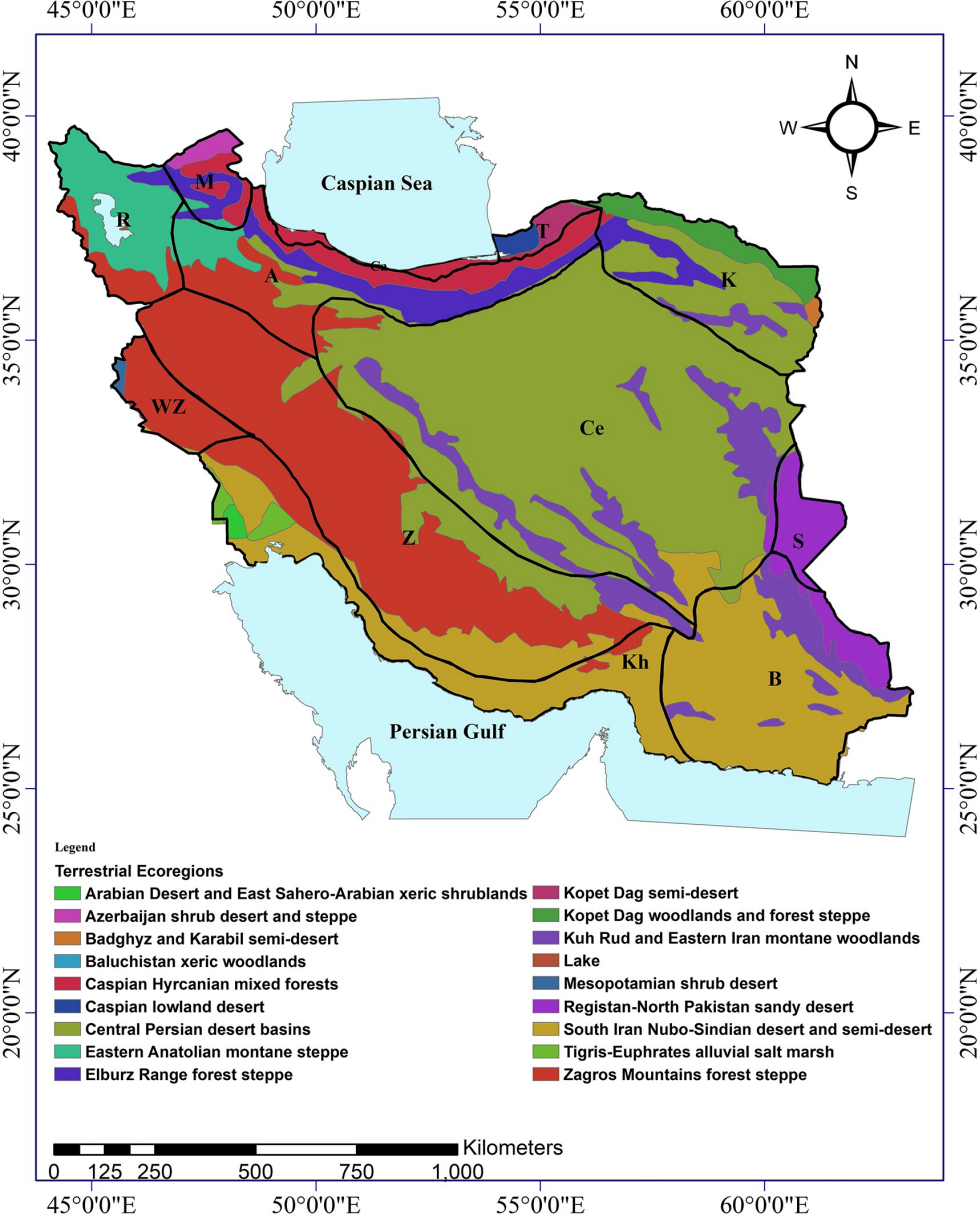
Map of terrestrial ecoregions of Iran [34]. Map designed using ARCGIS 9.3. Ecoregion shape file available from https://www.worldwildlife.org/publications/terrestrial-ecoregions-of-the-world.

Iran spans 1,648,195 square kilometers and is divided into thirteen distinct physiographic regions based on Anderson’s classification system. While Anderson did not provide precise geospatial coordinates delineating the boundaries of each region, we used geographic information system (ArcGIS 9.3, Esri) to generate proposed regional borders aligned with Anderson’s textual descriptions. Each of these regions exhibits particular topographic features (Fig 2), ecoregions (Fig 3), soil texture classes [32], and temperature [33], precipitation [33], and climate classes [33]. Considering the physical and ecological maps of Iran, we refined the GIS-based delineations to optimally match his intended regional frameworks. However, some uncertainty remains about the precise demarcation of certain regional boundaries due to Anderson’s original publication lacking explicit verbal boundary definitions.

These regions include the Alborz Mountains (covering approximately 7.74% of Iran’s total area), the Caspian Region (0.58%), the Central Plateau (33.19%), Iranian Baluchistan and the Makran Coast (12.15%), the Khuzestan Plain and Persian Gulf Coast (8.21%), Kopet-Dagh (6.69%), Moghan Steppe (1.65%), Reza’iyeh (Urmia) Basin (4.33%), Sistan Basin (1.64%), Turkmen Steppe (0.77%), Western Foothills of the Zagros Mountains (2.14%), Zagros Mountains (20.8%), and Islands of the Persian Gulf (0.12%) (Fig 2).

### Data collection

The distribution data were extracted from multiple sources: 1) previously published literature and books; 2) online databases, including the Global Biodiversity Information Facility (GBIF) [35, 36]; 3) herpetological collections of several zoological museums and institutes; and 4) occurrence data collected by the authors and their colleagues during numerous field surveys conducted in recent years. A total of 1,550 data points were extracted, all of which, except those recorded in GBIF, are presented in S1 Table with a spatial accuracy of about 10 km.

### Data limitation

Approximately 50% of the taxonomic distribution data lacked precision, precluding the possibility of assigning accurate geographic coordinates. Given the extensive size of the ecoregions and regions, the presence or absence of taxa within each was denoted by a binary system (1 or 0). The taxonomic locations within the ecoregions were established by aligning the regions polygons with the ecoregions map (Fig 3). However, data points situated near the borders of ecoregions or regions were excluded from the analysis due to the inherent imprecision of these boundaries. Consequently, the subsequent analyses were conducted based solely on the binary presence/absence data of the taxa.

### Measuring similarity among regions

In this analysis, we utilized a data frame that provides detailed information on the presence and absence of various species and subspecies across thirteen distinct regions and six surrounding areas described by Sindaco *et al*. [22].

In order to compare the similarity among the regions and surrounding areas, we performed Hierarchical Cluster Analysis utilizing Jaccard’s and Sorenson’s binary similarity indices and UPGMA linkage for cluster formation [37]. Both indices quantify the similarity between two regions based on presence-absence data at the genus and species (and subspecies) levels separately. Calculation of the Jaccard binary similarity matrix was done using the “vegdist” function in the R package “vegan” [38]. The Sorenson index matrix was computed in the R packages “vegan” and “dendextend” [38, 39].

### Indicator species (composition indicator)

Simplified Indicator Species analysis (ISA) [40] was used to detect the composition indicators [41]. We utilized a data frame that provides detailed information on the presence and absence of various species and subspecies across thirteen distinct regions and sixteen ecoregions. Initially, we performed a non-hierarchical clustering of the regions based on their ecoregions using the k-means clustering technique. This process resulted in the formation of five distinct cluster groups.

Following this, we conducted an ISA on the presence-absence matrix, which contained the occurrences of species and subspecies within each region. For this analysis, we employed the ‘indicspecies’ package in R 4.3.1 [42]. The cluster groups that were previously defined were used as the grouping factor in this analysis.

We utilized the ‘multipatt’ function to compute the Phi coefficient of association values for each species within each group. The Phi coefficients can range from -1 to 1. Positive values indicate that a species is associated with a group, and higher values signify a stronger association.

To test the statistical significance of the Phi coefficients, we performed a Monte Carlo test with 999 permutations. Species that had statistically significant Phi values greater than 0.7 were considered as characteristic species for their respective regional cluster groups [43].

This methodology allows for the identification of characteristic species for each regional cluster, based on species fidelity. The ISA provides an objective method to determine which species can be considered as composition indicator [41] of certain regional groupings, defined by similarities in their ecoregions.

### Measuring species richness and endemism

Species richness is quantified as the total number of species present in each region. This measure is fundamental to understanding biodiversity within a given area. For endemism, we initially identified range-restricted species per region. However, this measure of endemism presented two issues. Firstly, it exhibited a correlation with species richness, and secondly, it imposed an arbitrary limit on endemism [44].

To address these issues, we adopted a weighted endemism measure (WE), which involves counting all species in each region, irrespective of their distribution, and assigning each a weight that is the inverse of its range [44–47]. This measure allows us to account for the distribution of each species, providing a more comprehensive view of endemism.

The sum of these weights for all species in a region gives the region’s endemism score (WE). However, as this measure also correlated with species richness, we sought a measure of endemism that minimized this relationship. We achieved this by creating a Corrected Weighted Endemism (CWE) index, which is the weighted endemism index divided by the total species count in the region. This index, which measures the proportion of endemics in a region, showed minimal correlation with species richness in our data [44].

This approach allowed us to focus on deviations from the general relationship between species richness and endemism, which are of particular interest in endemism studies [48, 49]. It is important to note that while this method provides a more nuanced understanding of endemism, it may be influenced by the completeness and accuracy of the species distribution data. Summary of the steps are mentioned below:

*Data Collection*: The data frame, referred to above as ‘Indicator species’, was utilized in this analysis.

*Range Size Calculation*: Next, we calculated the range size for each species, which is the total number of ecoregions in which a species is found, irrespective to regions.

*Reciprocal Range Size Calculation*: We then calculated the reciprocal of the range size for each species. This was done by dividing 1 by the range size.

*Weighted Endemism Calculation*: The Weighted Endemism (WE) for each region was calculated by summing the reciprocal range sizes for all species present in that region.

*Corrected Weighted Endemism Calculation*: Finally, we calculated the Corrected Weighted Endemism (CWE) for each region by dividing the WE by the total number of species in that region.

### Historical biogeography

In this study, we conducted an extensive review of existing literature on the phylogeography of Iranian snakes. We sourced articles from various databases, ensuring a wide range of perspectives and findings. Our selection criteria included relevance to the topic, credibility of the source, and recency of the publication. Each article was thoroughly read and analyzed to extract key information about the speciation and dispersal patterns of taxa. We then synthesized this information to create a cohesive summary. This methodology allowed us to present an accurate and detailed overview of the subject matter, contributing to the existing body of knowledge on the biogeography of Iranian snakes.

## Results

The most recent taxonomic checklist includes species, subspecies, and certain clades or morphs. These taxonomic units are based on the latest presence and distribution data and are summarized in Table 1. Additionally, we examine the presence and absence of each taxon within the regions and areas delineated by Sindaco et al. and Anderson. These regions encompass both Iran and its surrounding areas. For biogeographical context, we provide detailed information about the biogeographic characteristics of Anderson’s 13 regions. This includes specifics on climate types, primary biomes, and ecoregions, which are presented in S2 Table.

**Table 1:** Up-to-date checklist of snakes recorded from Iran.

Taxa	Physiographic regions																		
Leptotyphlopidae Stejneger, 1892	Sa	O	W	Tu	Ar	Ir	Ce	Ca	A	M	R	Z	WZ	Kh	T	K	S	B	I
*Myriopholis blanfordii* (Boulenger, 1890)		*	*			*						*		*			*		
*Myriopholis hamulirostris* (Nikolsky, 1907)			*									*							
*Myriopholis macrorhyncha* (Jan, 1860)	*		*		*	*			*			*	*	*				*	
**Typhlopidae Merrem, 1820**																			
*Indotyphlops braminus* (Daudin, 1803)														*					*
*Xerotyphlops luristanicus* Torki, 2017			*									*							
*Xerotyphlops vermicularis* (Merrem, 1820)			*	*		*	*	*	*	*	*	*	*	*	*	*			
**Erycidae Pyron, Reynolds and Burbrink, 2014**																			
*Eryx elegans* (Gray, 1849)			*	*		*	*		*							*			
*Eryx jayakari* Boulenger, 1888					*									*					
*Eryx miliaris* (Pallas, 1773)				*		*	*		*						*	*	*	*	
*Eryx sistanensis* Eskandarzadeh, Rastegar-Pouyani, Rastegar-Pouyani, Zargan, Hajinourmohamadi, Nazarov, Sami, Rajabizadeh, Nabizadeh and Navaian, 2020						*								*			*	*	
*Eryx* sp. Eskandarzadeh	*		*						*	*	*	*	*	*					
**Colubridae Oppel, 1811**																			
*Boiga trigonata melanocephala* (Annandale 1904)			*	*		*	*								*	*	*	*	
*Coronella austriaca* Laurenti, 1768			*	*				*	*	*	*				*				
*Dolichophis andreanus* (Werner, 1917)			*			*						*	*	*					
*Dolichophis jugularis* (Linnaeus, 1758)			*	*				*	*		*	*	*		*	*			
*Dolichophis schmidti* (Nikolsky, 1909)			*	*				*	*	*	*	*	*		*	*			
*Eirenis collaris* (Méntériés, 1832)			*						*	*	*	*	*						
*Eirenis coronella* (Schlegel, 1837)					*								*	*					
*Eirenis coronelloides* (Jan, 1862)					*								*	*					
*Eirenis kermanensis* Rajabizadeh, Schmidtler, Orlov and Soleimani, 2012						*						*							
*Eirenis medus* Chernov, 1940			*			*			*			*				*			
*Eirenis modestus* (Martin, 1838)			*							*	*								
*Eirenis nigrofasciatus* (Nikolsky, 1907)			*									*	*	*					*
*Eirenis occidentalis* Rajabizadeh, Nagy, Adriaens, Avci, Masroor, Schmidtler, Nazarov, Esmaeili and Christiaens, 2016			*										*						
*Eirenis persicus* (Anderson, 1872)			*									*	*	*					*
*Eirenis* cf. *persicus* Rajabizadeh			*						*										
*Eirenis punctatolineatus* (Boettger, 1892)			*			*	*		*	*	*	*	*			*			
*Eirenis punctatolineatus condoni* (Boulenger, 1920)			*			*	*					*		*					
*Eirenis rafsanjanicus* Akbarpour, Rastegar-Pouyani, Fathinia and Rastegar-Pouyani, 2020						*						*							
*Eirenis rechingeri* Eiselt, 1971			*									*							
*Eirenis thospitis* Schmidtler and Lanza, 1990			*								*								
*Eirenis walteri* (Boettger, 1888)						*	*									*	*	*	
*Eirenis yassujicus* Fathinia, Rastegar-Pouyani and Shafaeipour, 2019			*									*							
*Elaphe dione* (Pallas, 1773)			*	*				*	*	*					*				
*Elaphe urartica**** ***Jablonski, Kukushkin, Avcı, Bunyatova, Kumlutaş, Ilgaz, Polyakova, Shiryaev, Tuniyev, Jandzik, 2019			*					*	*	*	*	*	*						
*Hemorrhois nummifer* (Reuss, 1834)			*	*				*	*	*	*	*	*		*	*			
*Hemorrhois ravergieri* (Ménétriés, 1832)			*	*	*	*	*	*	*	*	*	*	*	*	*	*	*	*	
*Lycodon bicolor* (Nikolsky, 1903)		*		*		*									*	*	*	*	
*Lytorhynchus gaddi* Nikolsky, 1907	*				*								*	*					*
*Lytorhynchus maynardi* Alcock and Finn, 1897						*											*	*	
*Lytorhynchus ridgewayi* Boulenger, 1887			*	*	*	*	*		*			*		*	*	*	*	*	
*Oligodon transcaspicus* (Nikolsky, 1902)			*	*											*	*			
*Persiophis fahimii* Rajabizadeh, Pyron, Nazarov, Poyarkov, Adriaens and Herrel, 2020						*						*							
*Platyceps atayevi* (Tuniyev and Shammakov, 1993)			*	*											*	*			
*Platyceps karelini* (Brandt, 1838)			*	*		*	*		*			*			*	*	*	*	
*Platyceps karelini chesneii* (Martin 1838)	*		*		*							*	*	*					*
*Platyceps mintonorum* (Mertens, 1969)						*											*	*	
*Platyceps najadum* (Eichwald, 1831)			*					*	*	*	*								
*Platyceps najadum albitemporalis* (Darevsky and Orlov, 1994)			*							*									
*Platyceps rhodorachis* (Jan in De Filippi, 1865)			*	*	*	*	*		*			*	*	*	*	*	*	*	*
*Platyceps* cf. *r*. *rhodorachis* Schätti, 2014^a^			*								*								
*Platyceps schmidtleri* (Schätti and McCarthy, 2001)			*			*						*	*	*			*	*	
*Platyceps ventromaculatus* (Gray, 1834)		*				*												*	
*Rhynchocalamus levitoni* (Torki, 2017)			*									*	*						
*Rhynchocalamus satunini* (Nikolsky, 1899)			*						*		*	*	*	*					
*Spalerosophis diadema cliffordii* (Schlegel, 1837)	*				*								*	*					*
*Spalerosophis schirasianus* (Jan, 1863)			*	*		*	*		*			*			*	*	*	*	*
*Spalerosophis microlepis* Jan, 1865			*				*					*	*						
*Telescopus fallax iberus* (Eichwald, 1831)			*					*	*	*	*	*	*						
*Telescopus nigriceps* Ahl, 1924			*									*	*						
*Telescopus rhinopoma* (Blanford, 1874)						*	*		*			*				*	*	*	*
*Telescopus tessellatus tessellatus*(Wall, 1908)			*				*		*			*	*						
*Telescopus tessellatus martini* Schmidt, 1939			*		*							*	*	*					
*Zamenis hohenackeri* (Strauch, 1873)			*						*	*	*	*							
*Zamenis longissimus* (Laurenti, 1768)			*					*	*	*	*	*							
*Zamenis persicus* (F. Werner, 1913)			*					*	*	*					*	*			
**Natricidae Bonaparte, 1838**																			
*Natrix natrix scutata* (Pallas, 1771)			*					*	*	*	*				*				
*Natrix tessellata* (Laurenti, 1768)			*	*	*			*	*	*	*	*	*	*	*	*			
**Psammophiidae Bourgeois, 1968**																			
*Malpolon insignitus fuscus* (Fleischmann, 1831)	*		*						*	*	*	*	*	*		*			
*Psammophis lineolatus* (Brandt, 1838)			*	*	*	*	*		*			*	*	*	*	*	*	*	
*Psammophis schokari* (Forskål, 1775)	*		*		*	*	*		*			*	*	*	*	*	*	*	
*Rhagerhis moilensis* (Reuss, 1834)	*				*									*					*
**Elapidae Boie, 1827**																			
*Bungarus persicus* Abtin, Nilson, Mobaraki, Hosseini and Dehgannejhad, 2014		*				*								*				*	
*Naja oxiana* (Eichwald, 1831)			*	*		*	*								*	*	*		
*Walterinnesia morgani* (Mocquard, 1905)					*								*	*					*
**Viperidae Oppel, 1811**																			
*Gloydius caucasicus* (Clade i)^b^ = Kopet Dagh-Eastern Alborz			*	*					*							*			
*Gloydius caucasicus* (Clade ii) = Lar National Park-Central Alborz			*					*	*										
*Gloydius caucasicus* (Clade iii) = Central Alborz			*					*	*										
*Gloydius caucasicus* (Clade iv) = Western Alborz-Azerbaijan			*						*	*									
*Cerastes gasperettii* Leviton and Anderson, 1967					*									*					
*Echis carinatus sochureki* Stemmler, 1969		*			*	*	*					*		*		*	*	*	*
*Eristicophis macmahoni* Alcock and Finn, 1897						*											*		
*Macrovipera lebetina chernovi* (Chikin and Szczerbak, 1992)			*	*											*	*			
*Macrovipera lebetina obtusa* (Dwigubskij, 1832)			*			*	*		*	*	*	*	*						
*Macrovipera razii* Oraie, Rastegar-Pouyani, Khosravani, Moradi, Akbari, Sehhatisabet, Shafiei, Stümpel and Joger, 2018			*		*	*	*					*	*	*					
*Montivipera kuhrangica* Rajabizadeh, Nilson and Kami, 2011			*									*							
*Montivipera latifii* (Mertens, Darevsky and Klemmer, 1967)			*						*										
*Montivipera raddei* (Boettger, 1890)			*							*	*	*							
*Montivipera raddei albicornuta* (Nilson and Andrén, 1985)									*										
*Montivipera wagneri* Nilson and Andrén, 1984			*								*								
*Pseudocerastes persicus* (Duméril, Bibron, and Duméril, 1854)			*		*	*	*					*	*	*		*	*	*	
*Pseudocerastes urarachnoides* Bostanchi, Anderson, Kami, and Papenfuss, 2006			*										*						
*Vipera eriwanensis* (Reuss, 1933)			*							*	*								
*Vipera eriwanensis ebneri* Knoepffler and Sochurek, 1955			*						*		*								

Distribution of each taxon in zoogeographical regions is based on Sindaco *et al*. [50], and Anderson [24]. Realm: *O*- Oriental, Regions: *Sa*–Saharan; *W*—Western Asian Mountains transition zone; *Tu*–Turanian; *Ar*–Arabian; Ir–Iranian, and subregions (Provinces): *Ce*—Central Plateau; *Ca*—Caspian; *A*—Alborz Mountains; *M*—Moghan Steppe; *R*—Reza’iyeh (Urmia) Basin; *Z*—Zagros Mountains; *WZ*—Western Foothills of the Zagros Mountains; *Kh*—Khuzestan Plain and the Persian Gulf Coast; *T*—Turkmen Steppe; *K*—Kopet-Dagh; *S*—Sistan Basin; *B*—Iranian Baluchistan and the Makran Coast; *I*—Islands of the Persian Gulf.

^a^Based on Schätti’s study, the populations from NE Iraq to S Urmia were grouped separately due to their morphological differences, such as a higher count of ventral plates [51]

^b^Based on phylogenetic study by [52].

## Historical biogeography

This text synthesizes available research to outline current understanding and emerging insights into Iranian snake phylogeography across diverse families encompassing all major biogeographic regions. It reveals the dynamic nature of Iranian snake distributions through time, and the critical roles of both earth history events and climate shifts in driving diversification from the Miocene onwards. This phylogeographic summary provides a foundation for future studies to build upon using phylogenetic methods and expanded sampling, especially in understudied regions. It highlights the importance of an integrative approach combining multiple lines of evidence to elucidate the evolutionary histories of Iranian snakes and interpret their current diversity patterns.

### Family Leptotyphlopidae Stejneger, 1892

#### Genus *Myriopholis* Hedges, Adalsteinsson & Branch, 2009

The snake genus *Myriopholis* is distributed across Africa, the Arabian Peninsula, and southwest Asia. The majority of the species are found in sub-Saharan Africa, particularly in west, central, and east Africa. *Myriopholis* belongs to the tribe Myriopholini within the subfamily Leptotyphlopinae, which contains Old World leptotyphlopids. Molecular dating analyses suggest the Myriopholini lineage diverged from other Leptotyphlopinae around 84 Mya in the Late Cretaceous. The deepest-branching *Myriopholis* species sampled was *M*. *longicauda* from South and East Africa, indicating the genus may have originated in southern Africa. Other divergences date to around 60 Mya, suggesting dispersal of *Myriopholis* out of Africa into Arabia and Asia later in the Cenozoic after the initial radiation within Africa [53].

In Iran the genus is found on the southern slopes of the Central and Western Alborz Mountains, extending west to the Western foothills of the Zagros Mountains and encompassing the entire Khuzestan plain. Its distribution also includes all the lowlands of southern Iran, the Southern Zagros, and the coastal plains of Bushehr along the Persian Gulf coast, extending south of Jazmurian to the Makran coasts. It is also present in the southern parts of the Eastern Mountains in Baluchistan and the Zabul plain.

### Family Typhlopidae Merrem, 1820

#### Genus *Xerotyphlops* Hedges, Marion, Lipp, Marin & Vidal, 2014

The molecular phylogeny indicates that *Xerotyphlops* is an early diverging clade within Typhlopidae that appears to have originated and diversified in desert regions. *Xerotyphlops vermicularis* exhibits the widest distribution, with isolated and fragmented populations occurring across mediterranean and semi-arid regions in southeastern Europe, the Middle East, and north Africa. In Europe, it is found in Greece, Albania, Bulgaria, and European Turkey. These populations likely dispersed from southwest Asia. In the Middle East, its range extends from Turkey to Iran and the Arabian Peninsula, representing the core portion of its distribution. The core populations in the Middle East likely harbor the greatest genetic diversity. From here, range expansions occurred outward across North Africa and into southeastern Europe. In Africa, it occurs in coastal northwestern Africa from Morocco to Tunisia, possibly representing a range expansion from southwest Asia. The disjunct distribution between Europe, the Middle East, and Africa likely reflects range expansions during past wetter climates, followed by fragmentation as aridification intensified in the late Miocene and Pliocene [54–56].

In Iran the genus is found in the northeastern Kopet-Dagh mountains, the Gorgan Plain, and the southern Alborz Mountain slopes up to the Central Plateau’s northern borders. It also inhabits the northern Alborz Mountain slopes down to the Caspian Plain coastline, and the northwestern Azerbaijan highlands to the Moghan plain. In the Zagros Mountain range, its distribution extends north to the Central Mountains at the southern edge of the Central Plateau, and west to the rocky borders of the Khuzestan plain.

### Family Erycidae Pyron, Reynolds & Burbrink, 2014

#### Genus *Eryx* Daudin, 1803

Molecular phylogenies indicate that the genus *Eryx* originated in Africa and dispersed multiple times between Africa and Eurasia during the Miocene. At least three dispersal events between Africa and Eurasia occurred based on dated molecular phylogenies. The first dispersal was from Africa to Asia in the Middle Miocene, giving rise to the Asian clade. The second dispersal was from Africa to China in the Late Miocene, evidenced by the fossil *E*. *linxiaensis* which groups with the extant *E*. *colubrinus*. The third dispersal likely involved the spread of *E*. *jaculus* from Africa into Europe and Asia, though the exact timing remains uncertain. These intercontinental exchanges were likely facilitated by land bridges and aridification during the Miocene. Recent studies have revealed uncertainty regarding the taxonomic validity of *E*. *jaculus* and some related sand boa populations. The holotype in Egypt displayed morphological differences when compared to populations previously classified as this species from western Iran and Iraq. Substantial genetic divergence was also found between the Iranian and Iraqi populations. Some distinctions were noted in habitat preferences and morphological traits as well, implying that the western Asian populations potentially constitute new species separate from true *E*. *jaculus*. However, more sampling and analysis of topotypic Egyptian *E*. *jaculus* material is still required to define unambiguous species boundaries. For the time being, the western Iranian populations are provisionally labeled *Eryx* sp., while the Iraqi populations are designated *Eryx* cf. *jaculus* [57–59].

In Iran the genus is found in various regions of Iran, excluding the Caspian Plain and Central Plateau. Interestingly, each of the four taxa has selected one of the four corners of the country as their dominant geographical ranges.

### Family Colubridae Oppel, 1811

#### Genus *Boiga* Fitzinger, 1826

The phylogeny and biogeography of the genus *Boiga* indicate an Indochinese origin, with downstream dispersal events to islands and continental regions such as South Asia. The phylogeny supports *Boiga* as a monophyletic genus and identifies three major clades (A, B, C). *Boiga trigonata* is part of clade B, for which the ancestral range is reconstructed as either Indochina or Indochina+Africa. This suggests *B*. *trigonata* likely dispersed from Indochina to South Asia. No instances of reverse colonization were inferred for *B*. *trigonata*. Its presence in South Asia is consistent with the predominant downstream dispersal pattern observed in the genus [60].

The sole member of this genus in Iran is found in the semi-desert regions of the country’s eastern half. Its distribution spans from the Makran coast, around Jazmurian, to the southernmost areas of the Central Mountains. It extends northwards through the eastern regions of the Central Plateau, reaching as far as the northeastern Kopet-Dagh mountains and the Turkmen Steppe.

#### Genus *Coronella* Laurenti, 1768

The smooth snake genus *Coronella* exhibits both deep evolutionary divergences dating back to the Miocene/Pliocene epoch as well as more recent post-glacial population structure, particularly in southern peninsulas like Iberia. The genus has adapted to colder and drier climates over time, influencing its current biogeography. Phylogenetic studies indicate *C*. *austriaca* and *C*. *girondica* are sister taxa that diverged in the Late Miocene/Early Pliocene period around 5–10 Mya. Mitochondrial DNA analyses reveal that *C*. *austriaca* has three major genetic lineages: Western (Iberia), Central (Central Europe and Balkans), and Eastern (Caucasus). This structure reflects historical barriers like mountains. There is a reduction in genetic diversity from south to north within each lineage, consistent with post-glacial northward expansions. Iberian populations of *C*. *austriaca* exhibit strong phylogeographic structure, with distinct clades dating back to the Messinian Salinity Crisis (~5 Mya). This suggests the Iberian Peninsula was an important long-term glacial refuge. Overall, the genus displays both ancient divergences and more recent population structure, shaped by climate change and barriers like mountains over time [61–65].

*Coronella austriaca* in Iran is found in the northern regions, starting from the Azarbaijan mountains and Moghan Plain. Its distribution extends eastwards along the Caspian plain and the northern slopes of the Alborz Mountain range, reaching up to the Gorgan plain.

#### Genus *Dolichophis* Gistel, 1868

The whip snake genus *Dolichophis* is distributed across Europe, the Middle East, and Asia. It was previously classified within the genus *Hierophis*, but molecular phylogenetic studies have shown it represents a distinct evolutionary lineage. Phylogeographic analyses suggest an early divergence between European versus Middle Eastern/Asian species groups within *Dolichophis*. The European species form one distinct phylogenetic lineage, while the Middle East/Asia species comprise a separate closely related lineage. Biogeographically, *Dolichophis* likely originated and diversified within the Mediterranean region and arid zones of the Middle East and Asia. The morphological differences observed between the species groups may reflect local adaptations to their environments over time. Overall, *Dolichophis* exhibits phylogenetic structure consistent with an early split between European and Middle Eastern/Asian lineages, followed by localized diversification shaped by climate and geography [66, 67].

This genus is found in various regions of Iran, excluding the Central Plateau and Sistan and Baluchistan in the southeast. Two species are distributed in the northern half of Iran, spanning from the northwestern Azerbaijan Mountains and North Zagros, eastwards along the Alborz Mountain range and the Caspian plain, reaching the Gorgan plain and parts of Kopet-Dagh in northeast. The third species, endemic to the Zagros Mountains, extends from its southern slopes to the Persian Gulf coasts.

#### Genus *Eirenis* Jan, 1863

The dwarf snake genus *Eirenis* represents a specialized lineage descended from *Hierophis*-like ancestors that dispersed and diverged within the arid regions surrounding the Mediterranean basin. Within the *E*. *persicus* complex, the divergence of the subgenus *Pseudocyclophis* likely occurred around 16–18 Mya, corresponding to the isolation of the Iranian plateau from the Anatolian plateau. This suggests the lineage dispersed into the Iranian plateau across the Alborz mountains. Further divergence into western and eastern clades occurred around 10–13 Mya, corresponding to the collision of the Arabia-Eurasia plates and formation of the Zagros mountains. This provided new habitats for divergence of the lineages. Diversification and speciation within the clades corresponded with mountain formation events in the Zagros, Alborz, and Himalaya ranges during the Miocene-Pliocene epoch, which formed new habitats and climatic zones. Overall, the phylogeography of *Eirenis* reflects an initial dispersal into the Iranian plateau, followed by divergence and speciation driven by geological events like mountain formation over the past ~20 million years [68].

The genus, boasting the highest number of species in Iran, is generally found throughout the country, with the exception of the Caspian and Gorgan plains. While some species are observed only in their type localities and others have a restricted distribution, the genus as a whole exhibit a broad presence across Iran.

#### Genus *Elaphe* Fitzinger, 1833 and *Zamenis* Wagler, 1830

The genus *Elaphe* has a widespread distribution across the Palearctic, northern Oriental, Nearctic and northern Neotropical regions, occupying heterogeneous habitats including mountain forests, grasslands, deserts, and tropical forests. The genus likely originated in tropical Asia based on the basal position of species like *E*. *porphyracea*, suggesting an Asian origin. *Elaphe* dispersed from Asia to North America a single time via the Bering Land Bridge during the early Miocene, leading to the radiation of New World ratsnakes. In contrast, the genus dispersed from Asia to Europe multiple times, resulting in distinct lineages like *Zamenis* in Europe. However, basal *Zamenis* lineages are found in the Middle East (*Z*. *hohenackeri* and *Z*. *persica*) indicating east to west dispersal in the Old World. Overall, the evolutionary history of *Elaphe* reflects dispersal out of tropical Asia, colonization of new regions, and subsequent in situ diversification [69–71].

The genera *Elaphe* and *Zamenis* share overlapping distributions in Iran. Both are found in the northwestern Azerbaijan Mountains and the Moghan plain, extending to the Alborz Mountain range. They also inhabit the lowlands, including the Caspian and Gorgan plains. Furthermore, *Zamenis* extends south to the Central Zagros.

#### Genus *Hemorrhois* Boie, 1826

The colubrid snake genus *Hemorrhois* is distributed across northern Africa, the Iberian Peninsula, and western Asia. Molecular phylogenetic analyses have identified two primary clades within *Hemorrhois* corresponding to distinct geographic ranges: A western clade comprising *H*. *hippocrepis* and *H*. *algirus* found in northwest Africa and the Iberian Peninsula, and an eastern clade containing *H*. *ravergieri* and *H*. *nummifer* in central Asia. These two clades are separated by the arid regions of North Africa, suggesting that an ancient vicariant event fragmented the ancestral distribution. From a biogeographical perspective, *Hemorrhois* likely originated and initially diversified within Mediterranean coastal regions. The central Asian species represent a subsequent eastward dispersal and geographic isolation event. Overall, the phylogeography of *Hemorrhois* reflects an evolutionary history shaped by fragmentation of an ancestral coastal range, vicariance, and long-distance dispersal [66].

The genus exhibits a broad distribution across Iran, inhabiting various habitats. However, it has yet to be recorded in certain regions, including the Persian Gulf coast stretching from the Khuzestan Plain to Makran and the Jazmurian Basin, as well as the Lut Desert and the central areas of the Central Desert.

#### Genus *Lycodon* H. Boie in Fitzinger, 1826

The colubrid snake genus *Lycodon* has a broad distribution across South and Southeast Asia, ranging from India and Sri Lanka eastwards to southern China and Southeast Asia. The *L*. *aulicus* species group occurs in South Asia, including India, Sri Lanka, and parts of Central Asia. This group contains *L*. *striatus* and related species, and is thought to have originated in Southeast Asia based on the basal position of *L*. *jara* from Myanmar. Biogeographically, members of this group likely dispersed westwards along the Himalayan foothills into Central Asia and southwards into peninsular India and Sri Lanka. The *L*. *striatus* complex refers to a subgroup of similar-looking barred and striped species including *L*. *striatus*, *L*. *bicolor*, and related taxa. Molecular phylogenies reveal two main lineages within the *L*. *striatus* complex: *L*. *striatus* sensu stricto, distributed in eastern and central India and Sri Lanka, and *L*. *bicolor* occurring in Central Asia and Pakistan. *L*. *striatus* likely dispersed from mainland Asia into Sri Lanka during Pleistocene glacial periods when sea levels were lower, supported by the shallow genetic divergence between Indian and Sri Lankan populations. In contrast, *L*. *bicolor* originated in Southeast Asia and dispersed westwards into Central Asia, following river valleys and plains. It exhibits a pattern of clinal variation in coloration along elevation gradients. The two lineages abut but do not significantly overlap in northwestern India, with the Indus River potentially forming a barrier between them. Overall, the biogeography of *Lycodon* reflects an evolutionary history of dispersal, isolation, and diversification across South and Southeast Asia [72].

The sole member of this genus in Iran, *L*. *bicolor*, exhibits two distinct distributions in eastern Iran. In the southeast, it is found in the Zabol plain and the foothills of the Eastern Mountains in Sistan and Baluchistan. In contrast, its northeastern distribution includes the northeastern Kopet-Dagh Mountains, extending westward to the Gorgan plain.

#### Genus *Lytorhynchus* Peters, 1863

The colubrid snake genus *Lytorhynchus* consists of small, fossorial species inhabiting arid regions of Africa, the Middle East, and southwestern Asia. This genus likely originated in Arabia or northeast Africa, later dispersing into Southwest Asia. The species of *Lytorhynchus* have allopatric distributions, suggesting isolation in different arid zones permitted divergence. Overall, the biogeography of *Lytorhynchus* reflects an evolutionary history of origin in Africa/Arabia, dispersal into Southwest Asia, and allopatric speciation driven by isolation across arid habitats [66, 67].

Excluding two species that are confined to the southwest and southeast, the third species of this genus is widely distributed across Iran. However, it is notably absent from the highlands of the Alborz and Zagros Mountains, the northwestern Azarbaijan Mountains, and the Caspian Plain.

#### Genus *Oligodon* Blyth, 1860

The colubrid snake genus *Oligodon* is primarily distributed across tropical South and Southeast Asia, however two species extend into the Middle East and Central Asia: *O*. *russelius* and *O*. *taeniolatus*. The *O*. *taeniolatus* populations in Iran and Turkmenistan are genetically and morphologically distinct, representing a separate evolutionary lineage called *O*. *transcaspicus* restricted to the Kopet-Dagh Mountains. In contrast, *O*. *russelius* occurs further east in Pakistan and Afghanistan. *O*. *transcaspicus* likely diverged earlier in the Miocene or Pliocene compared to *O*. *russelius*, which exhibits less differentiation across South Asia. The high Hindu Kush Mountains and arid Central Asian deserts probably impeded east-west dispersal and range expansion. Suitable habitats like semi-arid shrublands may have facilitated dispersal during certain Plio-Pleistocene climate periods. Both species represent important biogeographic components in the otherwise Palearctic Middle and Central Asian herpetofauna. Their disjoint modern distributions reflect past climate fluctuations and geological changes impacting connectivity between South and Central Asia [73].

The sole representative of this genus in Iran inhabits specifically the northern edges of the northeastern Kopet-Dagh Mountains, and the Turkmen Steppe.

#### Genus *Persiophis* Rajabizadeh, Pyron, Nazarov, Poyarkov, Adriaens & Herrel 2020

The monotypic genus *Persiophis* contains the single species *Persiophis fahimii*, which is endemic to southern Iran. Molecular phylogenetic analyses place *Persiophis* in a clade with other colubrid genera found in southwestern Asia, including *Rhynchocalamus* and *Lytorhynchus*. *Persiophis* likely represents a relictual eastern lineage that is adapted to the arid mountains of Iran. The origin of *Persiophis* remains uncertain, but likely involves the isolation of an ancestral population within Iran’s extensive central mountain ranges at some point in the past 20 Mya. *Persiophis* may represent an ancient divergence in the region, or could reflect more recent dispersal from adjacent desert regions. Further research is needed to elucidate the evolutionary history and biogeography of this enigmatic snake lineage [67].

#### Genus *Platyceps* Blyth, 1860

The colubrid snake genus *Platyceps* is distributed across SE Europe, northern Africa, the Middle East, and western and central Asia. Molecular phylogenetic studies support *Platyceps* as a monophyletic genus. Within *Platyceps*, some phylogenetic structure corresponds to geographic distributions: *P*. *atayevi* and *P*. *najadum* from Armenia and SE Europe and western Asia respectively, form one clade, while *P*. *florulentus* and *P*. *rubriceps* from the Middle East form another. *P*. *karelini* and *P*. *rogersi* from western Asia are sister taxa, and *P*. *rhodorachis* from Turkmenistan occupies a basal position relative to the other species. This pattern suggests an early divergence between central Asian and Middle Eastern *Platyceps* species groups. Biogeographically, *Platyceps* likely originated in the arid regions of central Asia, later dispersing and diversifying into the Middle East. The phylogenetic proximity of central Asian species reflects their shared biogeographic history, while Middle Eastern species also share a close evolutionary relationship, having diversified within that region. Further phylogenetic and phylogeographic analyses within *Platyceps* could elucidate the timing and nature of diversification within this widespread genus [66, 67]. Members of this genus exhibit a widespread distribution across the entirety of Iran.

#### Genus *Rhynchocalamus* Günther, 1864

The snake genus *Rhynchocalamus* comprises small, secretive species endemic to the Middle East region. Phylogenetic and biogeographic evidence suggests the genus likely originated in the Levant, followed by range fragmentation leading to the current disjunct distribution pattern. Each species occupies a distinct geographic range separated by expansive desert habitats, likely due to niche constraints restricting them to rocky slopes and precluding dispersal across sandy deserts. The Miocene expansion of arid habitats in Arabia and North Africa probably caused vicariance events that isolated ancestral populations. Molecular dating estimates that the genus diverged from its sister clade *Lytorhynchus* around 26 Mya (Late Oligocene). Within *Rhynchocalamus*, initial diversification began in the Middle Miocene with the northern lineage *R*. *satunini* diverging approximately 15 Mya. Further speciation occurred in the Middle Miocene, separating *R*. *arabicus* and *R*. *dayanae* (formerly *R*. *melanocephalus*) around 9 Mya. *R*. *melanocephalus* diverged around 11 Mya and underwent additional radiation in the Pliocene around 3.5 Mya. Ongoing tectonic activity and marine barriers in the Middle East from the Oligocene onwards drove vicariance and allopatric divergence of these geographically isolated species. Quaternary climate changes causing expanding arid zones likely restricted species ranges to their current fragmented distributions [74–77].

The distribution of this genus in Iran is discrete, with records indicating its presence in isolated locations such as the northwestern Azarbaijan Mountains and the southern slopes of the Central Alborz Mountains. Nevertheless, its dominant distribution is concentrated in the Zagros Mountains, particularly the western foothills of the Zagros Mountains.

#### Genus *Spalerosophis* Jan, 1865

The colubrid snake genus *Spalerosophis* has a disjunct Saharo-Sindian distribution across arid regions of Afro-Arabia, the Irano-Turan region, and Indo-Pakistan. Phylogenetic analyses indicate the earliest diverging lineage is *S*. *microlepis* endemic to the Zagros Mountains of Iran, dating back to the early Miocene around 21.7 Mya. This suggests an Iranian Plateau origin for the genus. Subsequent cladogenic events coincided with major geological uplifts that likely drove vicariance and allopatric speciation. The Zagros Mountains separated western (*S*. *diadema cliffordii*) and eastern (*S*. *diadema schirasianus*) lineages approximately 16.7 Mya based on a substantial phylogenetic split, supporting their status as distinct species. Further east, the Sulaiman Mountains uplift segregated *S*. *arenarius* and *S*. *atriceps* around 13.3 Mya from ancestral populations in Afghanistan and Pakistan. These sister taxa diverged more recently during the late Miocene, around 7.9 Mya. The restricted range of *S*. *microlepis* in the Zagros highlands is attributed to isolation by surrounding inhospitable lowland deserts. In summary, the evolutionary history of *Spalerosophis* reveals a predominant role of mountain barrier formation and regional vicariance events across the Middle East in shaping biogeographic patterns and phylogeographic structure from an Iranian Plateau origin [78].

The distribution of this genus is widespread across Iran, with the exception of the Caspian plain and the northwestern Azarbaijan Mountains.

#### Genus *Telescopus* Wagler, 1830

The genus *Telescopus* likely originated in Africa based on phylogenetic analyses, with its distribution centered in arid regions across Africa, Arabia, the Levant, southeastern Europe, and western Asia. Two main dispersal events out of Africa occurred: one to Arabia in the Miocene, and another across the Tethys Sea to southeastern Europe/western Asia around the Oligocene-Miocene boundary. Phylogenetically, there is a deep split between a northern clade (*T*. *fallax*, *T*. *hoogstraali*, *T*. *nigriceps*) and the remaining southern, Afro-Arabian species. Diversification of the genus does not appear to be constrained by climatic niche conservatism, as there is no clear phylogenetic signal related to climatic niche characteristics. Overall, the current distribution of *Telescopus* reflects an initial African origin followed by two major dispersal events out of the continent along arid corridors, with climatic niches evolving flexibly during diversification [60, 79, 80]. Members of this genus exhibit a widespread distribution across the entire Iran.

### Family Natricidae Bonaparte, 1838

#### Genus *Natrix* Laurenti, 1768

Phylogenetic and phylogeographic studies show that the semiaquatic snake genus *Natrix* likely originated in the Late Miocene around the Mediterranean region and subsequently dispersed outward. For *N*. *tessellata*, the oldest lineages originated in southwest Asia, particularly Iran, in the Miocene. From this origin, it spread both westward (reaching the eastern Mediterranean, Balkans, and Central Europe) and eastward into Central Asia, dispersing along major river valleys and basins. Postglacial warming allowed northward expansions in Central Asia following river valleys into steppe habitats. In contrast, *N*. *natrix* originated around the Mediterranean and expanded outward after the last glacial maximum from southern refugia like the Balkans and Italy. Lineages spread across northern Europe as well as into central Europe, with additional colonization of northeastern Europe from the Caucasus and Fennoscandia from southern and eastern refugia. Range expansions followed postglacial warming and melting ice sheets, with secondary contact between lineages. Dispersal ability in *Natrix* is facilitated by utilization of aquatic habitats, but distributions are limited by dry regions, high elevations, and cold areas. Overall, phylogeographic patterns in *Natrix* reflect Miocene origins around the Mediterranean, range expansions from southern refugia after glacial retreats, and dispersal along river valleys and shorelines [81–85].

The genus has a widespread distribution in Iran. It inhabits all mountain ranges, including the northeastern Kopet-Dagh Mountains, both the northern and southern slopes of the Alborz Mountains, the northwestern Azerbaijan Mountains and the Moghan steppe, and extensive parts of the Zagros Mountains, excluding the southern slopes. Furthermore, its distribution encompasses the northern Caspian and Gorgan plains, as well as specific habitats within the Khuzestan plains.

#### Family Psammophiidae Bonaparte, 1845

The psammophiine snakes comprise one of the major early-diverging clades within Lamprophiidae. Molecular phylogenetic analyses reveal two primary clades: one containing the northern African and Eurasian genera *Malpolon* and *Rhamphiophis*, and another with the remaining sub-Saharan genera such as *Mimophis*, *Hemirhagerrhis*, and *Psammophis*. The basal position of *Malpolon* and *Rhamphiophis* suggests an ancestral distribution centered in northern Africa and west Asia for early psammophiine lineages, with subsequent diversification across sub-Saharan Africa. Phylogeographic structure corresponding to geographic barriers like the Great Rift Valley in sub-Saharan genera indicates allopatric divergence has played a role in diversification [86, 87].

#### Genus *Malpolon* Fitzinger, 1826

The genus *Malpolon* likely originated in Africa, potentially the Maghreb region of northern Africa, based on its inclusion within the largely African psammophiine clade and sister relationship with the monotypic genus *Rhagerhis moilensis*. Divergence dating estimates the initial split within *Malpolon* at 3.5–6 Mya between western and eastern African clades. From the Maghreb, *Malpolon* dispersed west into southwest Europe and east around the Mediterranean. The western clade *M*. *monspessulanus* appears to have invaded southwest Europe across the Strait of Gibraltar during the Pleistocene, while the eastern *M*. *insignitus* clade spread earlier over a prolonged period around the eastern Mediterranean and into southwest Asia, acquiring greater genetic diversity. Fossil evidence indicates an earlier Pliocene invasion of southwest Europe by *Malpolon*, later replaced by recent arrivals. Overall, *Malpolon* exhibits a pattern of dispersal out of Africa, with the Strait of Gibraltar periodically facilitating migration between Africa and Europe. Divergence dating suggests *M*. *insignitus* split from its sister species *M*. *monspessulanus* around 4.6–8 Mya, with significant genetic divergence supporting its probable status as a distinct genus adapted to more arid conditions [88–91].

The genus *Malpolon* is represented in Iran by a single species, which exhibits a wide distribution across various regions. Its range encompasses the northwestern Azerbaijan Mountains and Moghan Plain, extends along the southern slopes of the Alborz Mountain range, and reaches eastward to the northeastern Kopet-Dagh Mountains. This taxon is also prevalent throughout the majority of the Zagros Mountain range, spanning from the northern slopes proximal to the Central Mountains to the southern slopes abutting the Persian Gulf. Notably, this species has not been documented within the Khuzestan Plain. Conversely, the genus *Rhagerhis* exhibits a primary distribution within the Khuzestan plain. Its range extends to the easternmost boundaries of the Persian Gulf coasts, yet does not extend further eastward, with no documented sightings along the Makran coasts.

#### Genus *Psammophis* Boie, 1826

Within Africa, *Psammophis* exhibits substantial phylogeographic structure and genetic divergence between populations isolated in different refugia during past climate shifts. Diversification likely began in the Late Miocene-Pliocene, driven by aridification and fragmentation of mesic habitats. Geographic barriers like the Atlas Mountains and river valleys have also restricted gene flow, promoting divergence. In Asia, deep genetic divergence between *P*. *lineolatus* and *P*. *turpanensis* likely resulted from uplift of the Tian Shan Mountains ~6 Mya. Broad distributions of some species indicate use of dispersal corridors like river drainages to traverse barriers during mesic periods [92–95].

The genus is widely distributed across Iran, with the exception of the northern and northwestern regions. These excluded regions encompass the Caspian plain, the northwestern Azerbaijan Mountains, the Moghan plain, parts of the northern Zagros, and the northern slopes of both the Central and Western Alborz mountains.

### Family Elapidae Boie, 1827

#### Genus *Bungarus* Daudin, 1803

The genus *Bungarus* originated in Asia and contains around 15 species of kraits. The center of diversity is in tropical southern Asia, with the highest species richness in India and Southeast Asia. *Bungarus* likely diverged from other elapid snakes around 25–30 Mya during the Oligocene epoch. Several studies using mitochondrial DNA sequences have revealed cryptic diversity and uncovered independently evolving lineages within wide-ranging *Bungarus* species: Three major clades were found within *B*. *fasciatus* corresponding to Indo-Myanmar, Sundaic, and East Asian distributions. Three phylogenetic lineages are recognized within the *B*. *multicinctus* complex in Southeast Asia and southern China, elevating them to full species status: *B*. *multicinctus*, *B*. *candidus*, and *B*. *wanghaotingi*. Deep genetic divergences suggest additional cryptic diversity likely remains to be characterized within the *B*. *multicinctus* complex. *B*. *sindanus* shows divergence between northern (Pakistan, northern India, Nepal) and southern (peninsular India) lineages, estimated to have split around 6 Mya. Biogeographic barriers such as the Isthmus of Kra in Thailand have influenced the evolutionary differentiation between mainland and Sundaic (island Southeast Asian) populations. Cycles of connection and fragmentation of landmasses caused by sea level fluctuations (e.g., the Sunda shelf) during the Pleistocene epoch have shaped phylogeographic patterns within *Bungarus*. Drier climatic conditions forming barriers to dispersal (e.g., in central India) led to divergence between northern and southern lineages within *B*. *caeruleus*, *B*. *sindanus*, and possibly other species. Overall, the evolutionary relationships and taxonomy of *Bungarus* remain incompletely resolved. Integrative approaches combining morphological, ecological, and expanded molecular data will help clarify species boundaries and diversification processes within this medically significant snake genus [96–100].

The distribution of this genus extends westward, with its westernmost boundary reaching the coast of Makran in the southeast of Iran.

#### Genus *Naja* Laurenti, 1768

The genus *Naja* originated in Africa and consists of 4 subgenera: *Naja*, *Uraeus*, *Boulengerina*, and *Afronaja*. The typical subgenus *Naja* likely originated from a single invasion of Asia from Africa. *N*. *oxiana* has a western Asian distribution in the Trans-Caspian region across northeastern Iran, Turkmenistan, Uzbekistan, Tajikistan, Afghanistan, Pakistan, and northern India. Phylogenetically, *N*. *oxiana* forms a clade with other central/western Asian cobras: *N*. *kaouthia*, *N*. *sagittifera*, and *N*. *atra*. This suggests a relatively rapid expansion of *Naja* from eastern to western Asia. Within its Trans-Caspian range, *N*. *oxiana* does not show clear genetic differentiation or subdivision. This suggests rapid dispersal from eastern Iran westwards without major barriers, allowing panmixia. The divergence of *N*. *oxiana* from *N*. *kaouthia*/*N*. *sagittifera* was estimated around 3.2 Mya in the late Pliocene. This corresponds with the spread of more open, arid habitats in Asia, allowing westward expansion. Demographic analyses indicate *N*. *oxiana* underwent population expansion around 2 Kya in the late Holocene, likely facilitated by its range in open foothill habitats not impacted by Pleistocene glaciations [101–103].

The genus has a distribution in Iran that spans from the Eastern Mountains, located east of the Central Plateau, to the Gorgan City. Its range extends along the northeastern Kopet-Dagh Mountains and reaches the Eastern Alborz Mountains and Turkmen steppe.

#### Genus *Walterinnesia* Lataste, 1887

Phylogenetic analysis shows that *Walterinnesia* forms a monophyletic group within the cobra family Elapidae, based on mitochondrial DNA sequences. This supports *Walterinnesia* as a distinct evolutionary lineage. Within *Walterinnesia*, *W*. *aegyptia* and *W*. *morgani* are very closely related sister taxa. The genetic divergence between them is extremely small based on mitochondrial DNA. *W*. *morgani* was originally described from Iran, suggesting an eastward distribution, distinct from *W*. *aegyptia* in Africa/Arabia. However, the Saudi Arabian specimens identified genetically as *W*. *morgani* are virtually identical to *W*. *aegyptia*, indicating they are the same species. This suggests the biogeographic boundary between *W*. *aegyptia* and *W*. *morgani* may break down, and they should be treated as conspecific with a broad Middle Eastern/North African distribution. More sampling and genetic analysis is needed to confirm the range limits and evolutionary relationships between various populations currently assigned to *W*. *aegyptia* or *W*. *morgani* across the region. The broad distribution of *W*. *aegyptia* reflects the desert/arid-adapted ecology of the species, allowing it to span Northern Africa and the Arabian Peninsula [104].

The distribution of desert cobra in Iran begins from the western foothills of the Zagros Mountains and the Khuzestan plain. It extends along the southern slopes of the Zagros Mountains and the coasts of the Persian Gulf, reaching as far as the northwestern regions of the Jazmurian depression located in southern Kerman Province.

### Family Viperidae Oppel, 1811

#### Genus *Gloydius* Hoge & Romano-Hoge, 1981

The genus *Gloydius* (Asian pit vipers) has a broad distribution across northern Asia, ranging from Europe in the west to China in the east. Phylogenetic analyses indicate that during the Pleistocene glacial periods, *Gloydius* populations expanded out of the eastern Palearctic region towards more central areas and lower latitudes, tracking cooler climates. One member of this genus, *G*. *caucasicus*, is distributed across northeastern to northwestern Iran and southern Azerbaijan. Dated phylogenetic trees indicate that *G*. *caucasicus* diverged from its sister species *G*. *caraganus* around 1.89 Mya in the early Pleistocene. Further diversification within *G*. *caucasicus* itself initiated in the mid-Pleistocene, starting approximately 1.25 Mya. This resulted in four primary mitochondrial lineages that are spatially distributed from northeastern to northwestern Iran. The divergence of these lineages was likely driven by cooling climates and glacial cycles during the Pleistocene, which restricted gene flow among populations leading to allopatric fragmentation. Their geographic distributions suggest isolation in multiple refugia along the Alborz Mountains. Phylogeographic analyses support the hypothesis that *G*. *caucasicus* dispersed from northeastern Iran westwards via the northern and southern slopes of the Alborz Mountains [52, 105].

The distribution of this genus extends westward, with its westernmost boundary reaching eastern Caspian Sea. This genus, represented by one species, spans the northern part of the Iran. It covers both low-altitude lands, including the Gorgan and Caspian plains, and the northeastern Kopet-Dagh Mountain ranges, as well as the Turkmen steppe. The distribution encompasses the entire Alborz Mountain range, including the northern and southern slopes, and extends to the Talesh Mountains, which mark the westernmost limit of its range.

#### Genus *Cerastes* Laurenti, 1768

The horned vipers of the genus *Cerastes* are distributed across northern Africa, the Sinai Peninsula, the Arabian Peninsula, and parts of the Middle East. Recent genomic evidence reveals an unexpected phylogenomic relationship within Cerastes, with *C*. *cerastes* and *C*. *vipera* recovered as sister species while *C*. *gasperettii* is more distantly related. This suggests *C*. *cerastes* likely originated in Africa, with later dispersal of an ancestral lineage into Arabia around 7 ± 3.83 Mya following increasing aridity during the late Miocene. Subsequent isolation and speciation of this Arabian lineage gave rise to *C*. *gasperettii*. Plio-Pleistocene climatic fluctuations around 4.22 ± 2.23 Mya then caused geographic isolation and speciation of the African ancestor into the current *C*. *cerastes* and the sand-specialist *C*. *vipera*. Within *C*. *cerastes*, genetic differentiation distinguishes several intraspecific lineages: northwestern African *C*. *c*. *cerastes*, northeastern African *C*. *c*. *cerastes* (Chad, Egypt), and Arabian *C*. *c*. *hoofieni* dated around 1.70 ± 0.92 Mya. For *C*. *gasperettii*, phylogeographic structure separates northern (Iran, eastern Arabia) and southern (Yemen, Oman) clades. However, relationships within *C*. *gasperettii* are not fully resolved due to limited sampling. Overall, the biogeographic pattern suggests an older African *C*. *cerastes* lineage that later dispersed into Arabia, isolating *C*. *gasperettii*. Subsequent aridification facilitated *C*. *gasperettii*’s rapid range expansion, while geographic barriers (e.g. Red Sea) and climatic shifts likely drove vicariance and divergence within *C*. *cerastes*. Secondary contact between divergent lineages probably occurred during range expansions [106–110].

A single species of this genus exhibits a restricted distribution, confined to the Khuzestan plain, southwestern Iran.

#### Genus *Echis* Merrem, 1820

The saw-scaled vipers of the genus *Echis* exhibit a widespread distribution across Africa, Arabia, and South/Southwest Asia. Phylogenetic analyses have revealed four main clades within *Echis* that largely correspond to geographic regions: the *E*. *ocellatus* group in West Africa; the *E*. *pyramidum* group in Africa north of the equator and Arabia; the *E*. *coloratus* group in Arabia; and the *E*. *carinatus* group in Southwest and South Asia. This disjunct distribution reflects the complex biogeographic history of Africa and Eurasia colliding in the early Miocene, followed by vicariance events like the opening of the Red Sea. Genetic divergence between the main clades generally reflects their geographic separation. The West African *E*. *ocellatus* group is the most divergent, indicating a long history of isolation. Within the *E*. *pyramidum* group, African and Arabian populations form deeply divergent sister clades, likely due to vicariance from the late Miocene opening of the Red Sea. Arabian members of this group show further subdivision, with divergence between *E*. *khosatzkii* in Oman and other taxa in Yemen. The Arabian *E*. *coloratus* group contains the closely related but deeply divergent taxa *E*. *coloratus* and *E*. *omanensis*, separated by arid lowlands in Eastern Arabia. In contrast, the broad-ranging *E*. *carinatus* group of South/Southwest Asia exhibits very low genetic divergence, suggesting rapid and recent dispersal northward from an origin in southern India. Populations across the northern part of the *E*. *carinatus* range (Pakistan, Iran, Middle East) are almost identical, while more divergent haplotypes originate from southern India. This points to a range expansion northward into southwest Asia likely occurring in the late Pliocene or Pleistocene [107, 109–112].

This genus has an extensive distribution in Iran. It is found in the northeastern Kopet-Dagh Mountains (excluding the Turkmen steppe), the Central Plateau, and the Central Mountain. Its range also covers Sistan and Baluchistan, Makran, and the coasts of the Persian Gulf extending to the Khuzestan Plain. Additionally, this genus is distributed throughout the Zagros Mountains, with the exception of the extremely cold heights of the Central Zagros.

#### Genus *Macrovipera* Reuss, 1927

The viper genus *Macrovipera* is distributed across North Africa, the eastern Mediterranean islands, the Levant, and eastwards into Asia as far as Kashmir. The center of diversity is in Asia, where species inhabit foothill, mountain, semi-desert, and steppe habitats. Tree species are widely recognized, *M*. *lebetina*, *M*. *razii* and the range-restricted *M*. *schweizeri* of the western Cyclades islands (Greece). *M*. *lebetina* is the predominant *Macrovipera* species in Asia and has several described subspecies across its range. Phylogenetic analysis of Iranian *Macrovipera* using the cytochrome b gene revealed two major clades: Clade A represents *M*. *razii*, endemic to Zagros Mountains. Clade B comprises two subclades—B1 in western/northwestern Azarbaijan and Alborz Mountains (*M*. *l*. *obtusa*) and B2 in northeastern Kopet-Dagh Mountains (*M*. *l*. *chernovi*). The divergence of *M*. *razii* around 10.5 Mya coincided with Zagros Mountains uplift. *M*. *l*. *obtusa* shows high haplotype diversity, suggesting persistence in northwestern Iran during Pleistocene glacial-interglacial cycles, characteristic of a glacial refuge. The discovery of the Iranian endemic *M*. *razii* highlights the role of geological and climatic events in shaping *Macrovipera* biogeography and phylogeography. Further research is still needed to fully elucidate the evolutionary history of this genus [109, 110, 113].

#### Genus *Montivipera* Nilson, Tuniyev, Andrén, Orlov, Joger & Herrmann, 1999

The viper genus *Montivipera* is endemic to mountainous regions of the Near and Middle East, distributed from eastern Greece, western Turkey, Lebanon, Syria and Armenia to central Iran. This genus contains several rare and endangered species with small, isolated populations. Phylogenetic analyses indicate *Montivipera* diverged from *Macrovipera* around 12–15 Mya during the Miocene, coinciding with Anatolian-Iranian plateau uplift and formation of the mountain chains they now inhabit. Two major clades are recognized: the *xanthina* complex in western Anatolia and the Levant, and the *raddei* complex in eastern Anatolia, the Caucasus, and Iran. These likely diverged due to tectonic movements separating populations. Diversification and speciation within *Montivipera* occurred mainly in the Pliocene and Pleistocene, driven by climatic oscillations that caused range fragmentation and isolation in montane refugia, resulting in allopatric divergence. Distribution modeling shows *Montivipera* habitats have shifted to higher elevations since the Last Glacial Maximum around 21,000 years ago, indicating climate change has been a major influence. Suitable habitats are now highly fragmented and isolated on mountaintops, containing genetically distinct populations. Many unknown isolated populations likely exist. Phylogeographic patterns show association with historical plant refugia in regional mountains, suggesting common effects of past climate changes [114–117].

The members of this genus in Iran exhibit a restricted distribution, confined to the highlands of the Alborz Mountains, the northwestern Azerbaijan Mountains, and the Central Zagros Mountains.

#### Genus *Pseudocerastes* Boulenger, 1896 and *Eristicophis* Alcock & Finn, 1896

*Pseudocerastes* and *Eristicophis* likely originated in the Arabian Peninsula. Ancestral populations were probably located in western Arabia, as the basal mitochondrial and nuclear gene lineages are found in *P*. *fieldi* from Israel, Syria and Iraq. Biogeographic reconstructions suggest an eastward dispersal of these genera from western Arabia onto the Iranian Plateau around 12–13 Mya (Miocene). This coincides with the formation of the Gomphotherium land bridge which likely facilitated trans-Arabian dispersal. The genus *Eristicophis* is currently restricted to the Registan–North Pakistan sandy desert, implying an isolation and speciation event in this eastern region. Within *Pseudocerastes*, *P*. *persicus* and *P*. *urarachnoides* likely diverged around 8 Mya (Miocene), potentially driven by the uplift of the Zagros Mountains acting as a vicariant barrier. Further lineage diversifications occurred more recently during the Pliocene and Pleistocene. Phylogeographic patterns reveal subdivision into an Arabian *P*. *fieldi* group and a Zagros/Iranian Plateau *P*. *persicus*/*P*. *urarachnoides* group, with isolation of populations by historical events like mountain uplift and climatic oscillations [118, 119].

Genus *Pseudocerastes* has an extensive distribution in Iran. It is found in the northeastern Kopet-Dagh Mountains (excluding the Turkmen steppe), the Central Plateau, and the Central Mountain. Its range also covers Sistan and Baluchistan, Makran, and the coasts of the Persian Gulf extending to the Khuzestan Plain. Additionally, this genus is distributed throughout the Zagros, with the exception of the extremely cold heights of the Central Zagros.

#### Genus *Vipera* Laurenti, 1768

Phylogeographic studies reveal Pleistocene climatic oscillations strongly shaped genetic diversity and distribution patterns within the genus *Vipera*. During cold glacial periods, cold-adapted species of the subgenus *Pelias* expanded their ranges, while warm-adapted species became isolated in Mediterranean peninsulas. This pattern reversed during warmer interglacial periods, leading to divergence between populations isolated in different refugia (e.g. Iberia, Italy, Balkans). Several contact zones likely represent secondary contact of formerly isolated lineages. However, climatic adaptations alone do not explain all phylogeographic patterns. Topography, habitat availability, and geographic barriers also play key roles. Some species show complex patterns, and there is evidence for local climatic adaptation driving genetic divergence in certain cases, with closely related species or lineages often occupying partially or fully distinct climatic niches. *V*. *eriwanensis* is part of the *’renardi* clade’, distributed in the Caucasus region and areas north/east of the Black Sea. Within this clade, *V*. *eriwanensis* forms a subclade with *V*. *ebneri* that diverged around 1.4 Mya. *V*. *ebneri* from northern Iran is probably just a subspecies of *V*. *eriwanensis*. *V*. *eriwanensis* occurs in Armenia, Nakhichevan, and northwestern Iran, inhabiting rocky habitats at moderate elevations. Recent genetic studies have found low divergence between *V*. *eriwanensis* and *V*. *renardi*, questioning its status as a distinct species. However, *V*. *eriwanensis* does show some private haplotypes in nuclear DNA and seems geographically isolated from *V*. *renardi*, and its status as a valid species is still accepted by many. Phylogeographic patterns suggest the Caucasus was an important Pleistocene refugia for *V*. *eriwanensis* and relatives, with range expansions during glacial periods [115, 120–122].

In Iran, the only species of this genus demonstrates a restricted distribution, showing a preference for the alpine steppe habitats found specifically within the highlands of the Alborz Mountains and the northwestern Azerbaijan Mountains.

## Ecological biogeography

Here we examined the ecological biogeography of snake taxa across Iran utilizing faunal similarity indices, indicator species analyses and measuring richness and endemicity. The aim was to elucidate biogeographical patterns and delineate regions of high snake diversity and endemism. The cladistic and similarity analyses assessed faunal relationships and clustering. Indicator species distributions further elucidated assemblage distinctiveness. Quantifications of richness and endemicity highlighted some regions. Eventually, the results reveal distinct faunal assemblages and centers of richness, with certain areas standing out as hotspots.

### Faunal similarity

**Fig 4:**
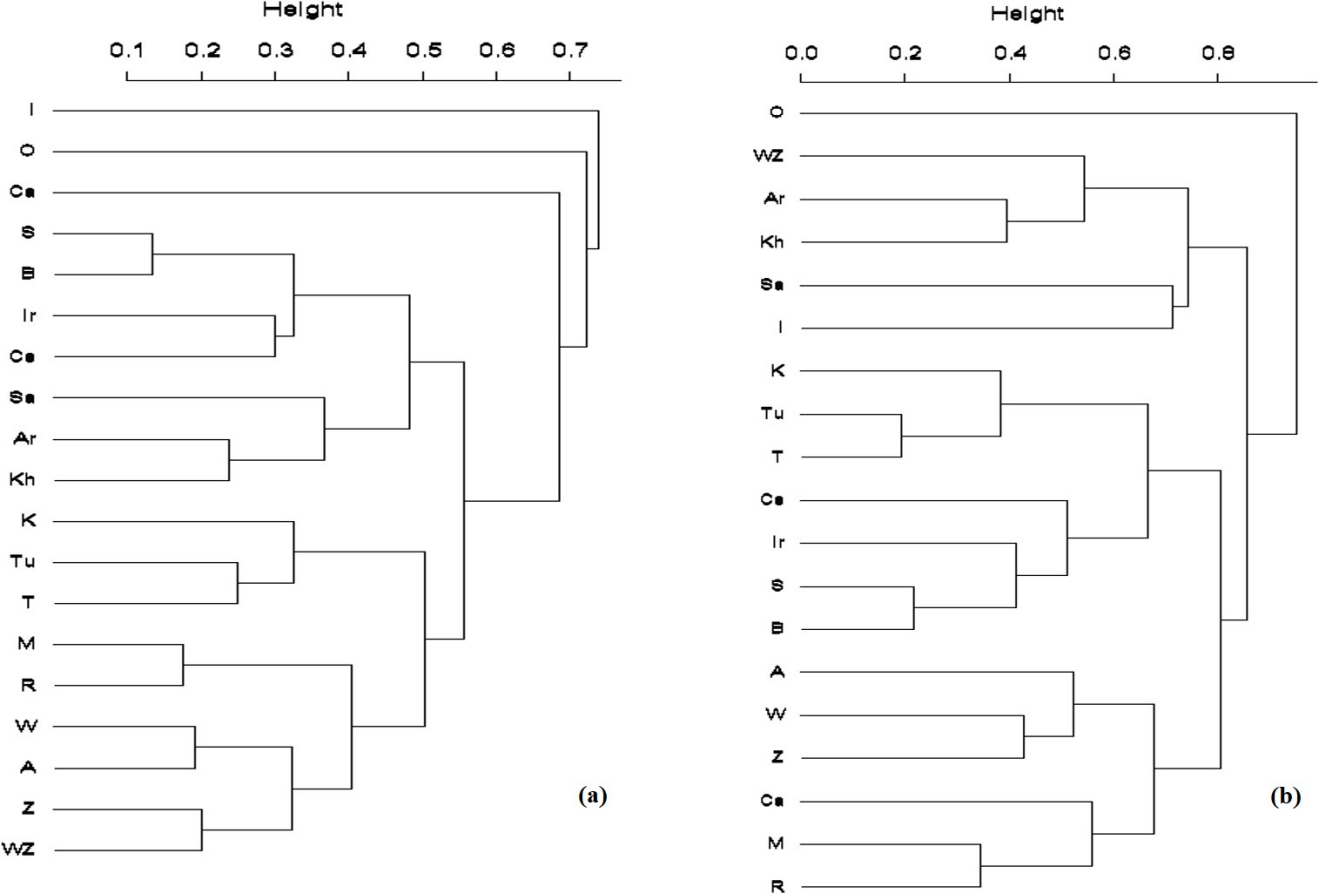
Similarity dendrogram using Jaccard index. (a) Generic Similarity, and (b) Species Similarity. Abbreviations as in Table 1.

The results of the cladistics analysis, based on both the Jaccard (Fig 4) and Sorenson methods (each independently considering the presence of genera and species), exhibited congruent outcomes. The pattern of area groupings remained consistent across both methodologies. The cladograms derived from the Sorenson method, along with the output matrices from the Jaccard method, are provided in S1 Fig. However, for the purpose of this discussion, we will focus solely on the results obtained from the Jaccard method, as depicted in Fig 4. The distance matrix of Jaccard similarity index between snake assemblages in Iran and surrounding regions for genera and species separately provided in S3 and S4 Tables respectively.

### Generic similarity

The dendrogram initially splits the southern Islands, which exhibit the least similarity to other regions (Fig 4A). Following this, the Oriental realm is distinguished, showing a high dissimilarity of 72.2% from the Western Palearctic. The third split separates the shoreline of the Caspian Sea from all remaining regions at a dissimilarity of 68.6%. The remaining regions are divided into two main groups based on shared and unique genera. The first group includes all southern and central regions, while the second group encompasses all highlands regions and the Turanian deserts. These groups are separated by a dissimilarity of 55.7%. Genera in the first group align with those in the southern Western Palearctic regions. In contrast, genera in the second group align with the northern Western Palearctic fauna. The first group further splits at a dissimilarity of 48.3% into southeastern (including the central plateau) and southwestern (including southern) subgroups. The southwestern subgroup shows the highest similarity to the Arabian and Saharan regions, while the southeastern subgroup is most similar to the Iranian region. The second group further splits at a dissimilarity of 50.2% into northeastern and the Alborz and Zagros Mountains subgroups. Genera in northeastern regions show the highest similarity to the Turanian region, while genera in the Alborz and Zagros Mountains show the highest similarity to the Western Mountain transition zone.

### Species similarity

The species and subspecies dendrogram distinctly separate the Oriental realm from the Western Palearctic with a dissimilarity of 95% (Fig 4B). Following this, the regions are divided into two groups. Group 1 includes the western, southwestern, and southern regions, while Group 2 encompasses all other areas. These groups are separated by a dissimilarity of 85.6%. Taxa in Group 1 align with those in the southern Western Palearctic regions. Group 2 further splits at a dissimilarity of 80.4% into two groups. The first group includes the Alborz and Zagros Mountains, as well as the shoreline of the Caspian Sea. Within this group, the Alborz and Zagros Mountains separate from the northwestern highlands and the shoreline of the Caspian Sea at a dissimilarity of 67.7%. Taxa in both these sets align with the Western Mountain transition zone region. The second group within Group 2 includes all of the lowland of Central Iran and Baluchistan, as well as the northeastern regions. This group further subdivides, with the northeastern region diverging from the central and southeastern areas at a dissimilarity of 66.6%. The taxa in the northeastern region show the highest similarity to the Turanian region, while the taxa in the central and southeastern areas are most similar to the Iranian region.

### Indicator species

Simplified ISA identified 31 species significantly associated with specific clusters or combinations of clusters (p<0.05). The clusters represent different regions: Cluster 1 = regions Ce, S, B; Cluster 2 = regions Ca, A, M, R; Cluster 3 = regions Z, WZ, Kh; Cluster 4 = regions T, K; Cluster 5 = region I. Seven species were indicators of individual clusters, including *Platyceps najadum* for cluster 2, *Dolichophis andreanus* and *Telescopus tessellatus martini* for cluster 3, and *Oligodon transcaspicus*, *Platyceps atayevi*, and *Macrovipera lebetina cernovi* for cluster 4. The three exclusive indicators for cluster 4 again suggest this cluster representing regions T and K has distinct ecoregions compared to other clusters. Seventeen species were indicators of pairs of clusters, with *Pseudocerastes persicus* and *Platyceps schmidtleri* for clusters 1+3, *Boiga trigonata melanocephala*, *Eryx miliaris*, *Eirenis walteri*, and *Lycodon bicolor* for 1+4, *Telescopus fallax* for 2+3, *Coronella austriaca*, *Zamenis persicus*, and *Natrix natrix* for 2+4, and *Eirenis nigrofasciatus*, *Eirenis persicus*, *Platyceps karelini chesneii*, *Lytorhynchus gaddi*, *Spalerosophis diadema cliffordii*, *Rhagerhis moilensis*, and *Walterinnesia aegyptia morgani* for 3+5. The large number of indicators for the 3+5 combination implies regions Z, WZ, Kh and I have substantial ecological similarities. Six species were indicators of 3 cluster combinations, including *Psammophis lineolatus* and *Psammophis schokari* for 1+3+4, and *Natrix tessellata*, *Dolichophis schmidti*, *Hemorrhois nummifer*, and *Xerotyphlops vermicularis* for 2+3+4. *Platyceps rhodorachis* was an indicator across all clusters 1+3+4+5, reiterating its role as an ecoregion generalist. The cluster with the highest number of exclusive indicator species remained cluster 4 (regions T, K) with 3 species. Clusters 1 (regions Ce, S, B) and 5 (region I) again had no exclusive indicators.

### Species richness and endemism

**Fig 5:**
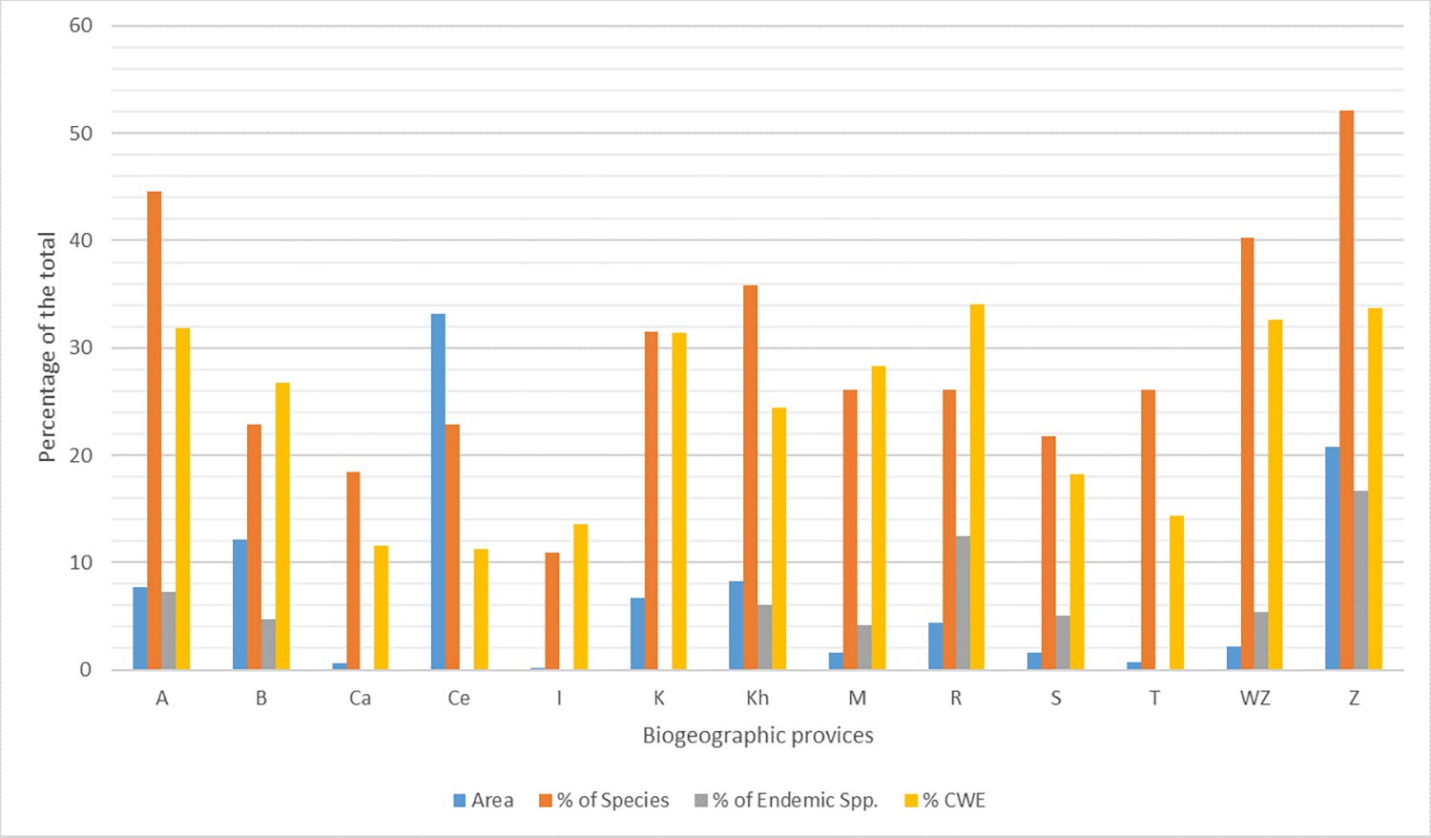
Percentage of the total species (richness), endemic species and percentage of CWE compared to area for each of the physiographical regions. Abbreviations as in Fig 2.

Analysis of the species distribution data across the defined regions revealed distinct patterns of species richness and endemism (Fig 5). The percentage representation of total species was highest in region Z (52.17%), followed by region A (44.57%). However, these two regions accounted for only 20.8% and 7.74% of the total study area, respectively. This indicates that regions Z and A are centers of high species richness. For endemic species representation, region Z again had the highest percentage with 16.66% of total endemic species, followed by region R with 12.5%. The CWE index, which accounts for total species and gives the proportion of endemic species per region, also identified Z and R as the top centers of endemism. Region R had the highest index value of 0.341, followed closely by region Z with a value of 0.337. In contrast, region Ce accounted for the largest percentage of the total study area (33.19%) but had low representations in terms of species and endemic species percentages. This suggests Ce is relatively poor in biodiversity compared to centers of richness and endemism. Some regions, including Kh, M, S and WZ, had moderate percentages of endemic species representation, between 5–6% of the total endemic species pool. This indicates they may be considered secondary centers of endemism, although not as significant as regions Z and R.

## Discussion

The delimitation of species boundaries has long been a contentious issue in taxonomy. Numerous species concepts have been formulated to account for the variation and differentiation between species groups [123], including the Morphological, Phylogenetic, and Ecological species concepts. The criteria used to determine sufficiently discrete species remain variable, with common benchmarks being morphological distinctiveness, reproductive isolation, ecological niche partitioning, and monophyly [124]. In this study, we advocate for retaining the rank of subspecies (and evolutionary lineages), as these intermediate designations highlight intriguing biogeographical and ecological phenomena related to the processes of dispersal and adaptation in closely allied taxa. This comprehensive approach provides a broader understanding of the biogeography of the taxa. Undoubtedly, there may be cryptic and undiscovered species or subspecies within this group. Our study represents an initial step, and future research by other scientists will further enhance our understanding.

### Penetration patterns and evolutionary origins

Previous phylogeographic analyses have revealed four primary gateways through which snake genera and species have penetrated and diversified in Iran, beyond endemic species that originated within the region [50]. 1. The Arabian region has facilitated the entrance of genera with Afrotropical origins such as *Myriopholis*, *Eryx*, *Telescopus*, and *Psammophis* [53, 57, 95, 80], Saharan origins like *Xerotyphlops*, *Lytorhynchus*, *Malpolon*, and *Cerastes* [55, 56, 66, 88, 108], and Arabian origins including *Rhynchocalamus*, *Walterinnesia*, and *Pseudocerastes* [76, 104, 118, 119]. These genera penetrated to the Iranian Plateau through the Arabian region in southwestern Iran [50]. 2. The Western Asian mountains transition zone has enabled the entrance of genera with northern Mediterranean origins such as *Coronella* and *Vipera* [63, 120] and eastern Mediterranean origins like *Dolichophis*, *Eirenis*, and *Hemorrhois* [66]. These genera likely spread into northwestern Iran through this mountain corridor [50]. Other genera including *Montivipera*, *Spalerosophis*, and *Macrovipera* and *Zamenis* may have originated within the Western Asian mountains transition zone [70, 78, 107, 113, 116]. 3. The Turanian region has facilitated the entrance of genera with Central Asian origins like *Platyceps*, *Gloydius*, and *Elaphe* into northeastern Iran [50, 52, 66, 71]. 4. The Indus River Valley has acted as an important biogeographic corridor and barrier between the Palearctic and Oriental realms. This region serves as a gateway for oriental genera to penetrate into the Iranian biogeographic region from the southeast and east, while also limiting the distributions of some Palearctic species [50]. Genera that have entered Iranian region via the Indus River Valley include *Boiga*, *Lycodon*, *Oligodon*, and *Bungarus* [50, 60, 72, 73]. Other genera such as *Echis* and *Naja* diversified in India after originating in Africa, with *Echis carinatus* and *Naja oxiana* later spreading into Iranian region across the Indus River Valley [103, 111]. Only the monotypic genera *Persiophis* and *Eristicophis* are endemic to the Iranian region.

### From ecoregional patterns to faunal distributions

The ecoregion similarity provides a good first approximation of faunal associations among Iran’s biogeographic regions. The Caspian forests, central deserts, Zagros woodlands, southern deserts, and northeastern semi-deserts contain distinct faunal assemblages adapted to these habitats. The generic dendrogram aligns fairly well with this ecoregion-based grouping, separating out Caspian, central, Zagros, southern desert, and northeastern generic faunas. However, some differences are apparent; generically the Alborz mountains group with the Zagros rather than the Caspian regions as predicted by the ecoregions. This suggests some faunal mixing between the Alborz and Zagros ranges [24]. At the species level, further complex patterns emerge. While the Zagros/Alborz, southern desert, and central/northeastern generic groupings are still discernible, the Zagros and Alborz mountains segregate into distinct species assemblages. Meanwhile, the central desert and northeastern regions show greater species overlap than expected generically, indicating more recent faunal mixing and diversification between these areas. Overall, while ecoregion similarity provides a broad picture, generic and especially species dendrograms reveal additional nuances of biogeographic affinity, divergence, and interchange among Iran’s faunal regions that reflect overlapping ecological, historical, and evolutionary processes.

### Ecoregional trends and refining by indicator species

The broad biogeographic patterns discerned from ecoregional associations are largely validated by indicator species analysis, though with finer resolution. The Caspian Hyrcanian mixed forests containing *Platyceps najadum*, central deserts lacking exclusives, Zagros woodlands without pure indicators but shared species with the Persian Gulf islands, and northeastern semi-deserts with the most exclusive indicators (3 species) align with these regions’ respective ecoregions. However, the Zagros and Alborz mountains segregated in the indicator analysis rather than grouping generically as in the ecoregional synthesis, congruent with their divergence at the species level. While widespread ecoregions showed an absence of distinct indicators, shared indicators between certain clusters matched habitat continuities, like the woodlands of the Zagros and Persian Gulf islands. Overall, the exclusive and shared significant indicator species generally confirm the broad ecoregional biogeographic patterns, but provide greater nuance and resolution. They align with generic homogenization of widespread ecoregions yet divergence between the Alborz and Zagros ranges. By combining ecoregional approximation with indicator species analysis, this multi-faceted biogeographic synthesis for Iran reveals both broad generalities and fine complexities in these patterns of faunal affinity and distribution.

Despite the presence of endemic species and subspecies in 13 regions, they often fail to serve as significant indicators due to several factors. These include e.g. limited sample sizes [125–127], specific habitat requirements [128, 129], residing in transitional zones [130], existence of cryptic species [131–133], dispersal abilities [134, 135], historical distributions [136], and sensitivity to environmental disturbances [137–139].

### Species richness and endemicity

Previous studies agree that the Zagros Mountains and adjoining areas, including the Khuzestan Plain and Western foothills of the Zagros Mountains, southwestern slopes of the Zagros Mountains, and Central Plateau, are hotspots of reptile species richness in Iran [20, 25, 26, 28, 140]. The highlands of this vast region fall within the Irano-Anatolian biodiversity hotspot and it is highlighted by all studies as having high total reptile richness. The Alborz Mountains and adjoining areas to the north and south are also identified by multiple studies as hotspots, as are areas of eastern Iran including the Kopet-Dagh Mountains and Sistan Basin [20, 25, 26, 140]. Some differences exist in the precise hotspot boundaries identified, but the core areas are consistent. Endemic species hotspots are concentrated in the Western Zagros foothills and central Zagros per some studies, while others find wider endemic richness [25, 28]. Hosseinzadeh *et al*. uniquely identified hotspots in Reza’iyeh Basin, Sistan Basin, and Iranian Baluchistan and Makran Coasts [25]. Yousefi *et al*. reported the highest total genetic divergence in areas around the Lut Desert in the southeast of Central Plateau and Jazmourian Plain in Iranian Baluchistan and the Makran coast, a finding not echoed in other studies [28].

Our findings suggest that the three regions of the Zagros (including Western foothills), Alborz Mountains, and Khuzestan Plain and the Persian Gulf coasts, identified as hotspots of snake biodiversity, exhibit a higher species richness compared to other regions within Iran. These findings align with those of previous studies. However, in contrast to some studies, the species richness in the Central Plateau and Sistan and Baluchistan Province is found to be lower than in other regions. The observed discrepancy can likely be attributed to the unique focus of this study on snake biodiversity. This focus is distinct from previous investigations, which have primarily concentrated on either lizard species or the broader scope of reptile biodiversity within Iran.

The high endemicity rate in the Zagros Mountains forest steppe ecoregion of Zagros Mountains and Eastern Anatolian montane steppe ecoregion of Reza’iyeh is attributed to isolation caused by geographic barriers and climate patterns that promoted endemic speciation over evolutionary timescales. This is aligning with findings from previous studies identifying these regions as hotspots for endemic reptiles in Iran [25, 26].

The uplifting of the Zagros Mountains around 22 million years ago opened up a wide range of previously unoccupied ecological niches and caused isolation from other parts of the country, providing conditions conducive for speciation [141]. The Zagros region can be considered not only a melting pot of different bioregions, as it represents a barrier and contact zone between the Mesopotamian fauna and the Central plateau [20, 24, 27, 142], but also a dispersal barrier and corridor for various lizard [e.g. 22, 24, 136] and snake species [e.g. 50, 68, 78, 113, 119]. The Zagros Mountains, characterized by high total genetic divergence, are recognized as glacial refugia during past climatic fluctuations [28, 143–146]. The region has also been identified as a hotspot of biodiversity for mammals and plants [10, 147]. Kafash *et al*. indicated that the Zagros Mountains have a combination of moderate climate stability and high topographic heterogeneity, providing diverse climatic niches [20]. Moreover, the Zagros Mountains are located within the Irano-Anatolian biodiversity hotspot [8, 9], demonstrating nested local hotspots within a regional hotspot [10]. The Zagros Mountains were also identified as an area of endemism for Western Palearctic reptiles [7].

The snake species *Xerotyphlops luristanicus*, *Eirenis rechingeri*, *E*. *yassujicus*, *Persiophis fahimii*, and *Montivipera kuhrangica* are all endemic to the Zagros Mountains forest steppe ecoregion. We disagree with Safaei *et al*. that *Pseudocerastes urarachnoides* and *Dolichophis andreanus* are also “endemic” to this ecoregion [148]. The spider tail viper has a wide distribution and stable populations in the ecoregion of the Mesopotamian shrub desert of the western foothills of the Zagros Mountains [119]. Regarding *Dolichophis andreanus*, a study identifying suitable habitats has confirmed Safaei’s theory [149]. However, based on the results obtained in this study and the distribution map presented in Rajabizadeh’s book [150], it has been determined that this species also has populations in the South Iran Nubo-Sindian desert and semi-desert ecoregion of the southern slopes of the Zagros Mountains to the Persian Gulf coasts. Except for *Eirenis rechingeri* and *Montivipera kuhrangica*. *Eirenis kermanensis* and *E*. *rafsanjanicus* inhabit the easternmost end of the Zagros in Kuh Rud and the Eastern Iran Montane woodlands ecoregion instead. This region is part of the fifth area of endemism for Iranian vascular plants, which contains 12% of Iran’s endemic species [151]. Interestingly, the monotypic genus *Persiophis* evolved in this region. *Myriopholis hamulirostris* is primarily found in the southern slopes of the South Iran Nubo-Sindian desert and semi-desert, and probably also the Zagros Mountains ecoregion. The narrow ranges and ecological niches of these species reflect long-term adaptation to these ecoregions. We also disagree with Safaei *et al*. that *Telescopus tesselatus* and *Walterinnesia* are “common” in the Zagros Mountains forest steppe [148]; our results suggest they are more common in the South Iran Nubo-Sindian desert and semi-desert ecoregion [152, 153].

The Corrected Weighted Endemism (CWE) index of a region is not solely determined by the number or percentage of endemic species present. It also takes into account the distribution of these species across the region [154]. In our study, we found that the Zagros Mountains, despite having a higher percentage of total and endemic species compared to Reza’iyeh Basin, had a lower CWE index. This can be attributed to the wider distribution of endemic species in the Zagros Mountains. If these species are found in more ecoregions within the region, it results in a lower weighted endemism index, thereby reducing the CWE [154].

Reza’iyeh Basin has the unique biomes Temperate Grasslands, Savannas and Shrublands, and the main ecoregion of this region is Eastern Anatolian montane steppe [34]. Reza’iyeh Basin have two climate types Arid, with cold season and Mediterranean, winter rains [155]. The extreme continental climate with hot, dry summers and cold winters supports open woodlands and alpine grasslands at high elevations. These habitats are suitable for endemic mountain vipers like *Montivipera wagneri* and *Vipera eriwanensis*. Furthermore, the Azerbaijan Plateau (except Moghan Steppe) has 21% of Iranian endemics which 11% of all Iranian vascular flora restricted only to this area. However, it has fewer plant endemics compared to Alborz Mountains despite being larger, likely due to less complex topography [151].

The Alborz Mountains has the second highest number of taxa after the Zagros Mountains (44.5%). The region contains the Alborz Range forest steppe ecoregion, which shares floral similarities with the surrounding highlands but has variations in rainfall and temperature. The region has the second highest number with 758 endemic plant species (29% of Iranian endemics) and 15% of all Iranian vascular flora are restricted only to the Alborz Mountains. Though smaller than Zagros, Alborz has a high concentration of endemics due to its wide elevational range and habitat heterogeneity [151]. Endemic vipers *Montivipera latifii* and *Vipera eriwanensis ebneri* reflect long-term isolation in the alpine steps of the Alborz Mountains [117, 148]. While the Alborz Mountains also contain a high diversity of habitats promoting endemism, the area is smaller and has less complex terrain compared to Zagros. Nevertheless, previous studies have highlighted the Alborz Mountains as an area of high reptile richness and genetic divergence for lizards [25, 28].

The Moghan Steppe has the Azerbaijan shrub desert ecoregion in the north, distinct from the southern woodland areas. The eastern lowlands contain Caspian Hyrcanian mixed forests, which share the endemic subspecies *Platyceps najadum albitemporalis* with the Caspian shoreline due to habitat similarities. The Moghan Steppe, along with the Caspian shoreline and northern slopes of the Alborz mountains, fall into the Caucasus plant biodiversity hotspot [151]. Another endemic species to the Caspian Hyrcanian mixed forest ecoregion is *Zamenis persicus*, however Safaei *et al*. mentioned this species as a “common species” in this ecoregion [148].

In the southeastern lowland areas, the Registan-North Pakistan sandy desert in Sistan and Baluchistan provides isolation for endemic sand-adapted species [24, 50] like *Eristicophis macmahoni*, *Lytorhynchus maynardi*, *Platyceps mintonorum* and *Eryx sistanensis*. However, Safaei *et al*. mentioned *Eryx sistanensis* and *Eristicophis* as “common species” for this unique ecoregion [148]. To the south, the South Iran Nubo-Sindian desert and semi-desert ecoregion in Iranian Baluchistan and the Makran Coast has high summer aridity, supporting distinct desert and xeric shrubland habitats [34]. Species endemic to this ecoregion like *Bungarus persicus* [148] and *Platyceps ventromaculatus* are adapted to the coastal semi-deserts.

In the opposite southwest direction, the Khuzestan Plain with some unique ecoregions and climate types is home to nearly 36% of the Iranian ophiofauna, adapted to the harsh climate. The sand-adapted species *Cerastes gasperettii* and *Eryx jayakari* are primarily endemic to the Arabian Desert and East Saharo-Arabian xeric shrublands ecoregion, but are also found in the South Iran Nubo-Sindian desert and semi-desert ecoregion. Other likely unique sand-adapted species in this region include *Spalerosophis diadema cliffordii* and *Lytorhynchus gaddi*. Safaei *et al*. mentioned *Cerastes gasperetti* and *Ragerhis moilensis* as "common species" of the Arabian Desert and East Saharo-Arabian xeric shrublands and the South Iran Nubo-Sindian desert and semi-desert ecoregions, respectively [148].

In contrast, the Central Plateau contains the Central Persian desert basins ecoregion, defined by lower elevation sandy deserts and shrublands with high aridity. There are fewer geographic barriers, allowing taxa like racers and whipsnakes to inhabit both the Central Persian desert basins and neighboring arid ecoregions. The lack of climate and terrain heterogeneity limits speciation and endemism compared to the surrounding highlands. Safaei *et al*. mentioned *Spalerosophis microlepis* as an "endemic species" and *Pseudocerastes persicus* as a "common species" in the Central Persian desert basins ecoregion [148]. However, our results disagree with these assessments.

## Conclusion

This first comprehensive investigation of snake biogeography in Iran integrates historical and ecological perspectives to elucidate complex patterns of affinity, divergence, and distribution. Penetration gateways enabled faunal interchange and range expansions from adjoining regions, facilitated by habitat continuities yet also delimited by geographic barriers. Isolation within Iran’s diverse terrain promoted endemic speciation, with richness concentrating in the Zagros and Alborz ranges. Ecoregional frameworks provide an approximation refined by indicator species analysis, revealing generalities underlying biodiversity gradients. Endemism hotspots highlight the interplay of past climate fluctuations and terrain heterogeneity in driving endemic diversity. The Zagros Mountains represent a melting pot shaped by its boundary dynamics, while Reza’iyeh Basin demonstrate nested endemism. Phylogeographic structuring points to cryptic diversity warranting further investigation. By synthesizing multi-faceted techniques, this research elucidates biogeographic trends, complexities, and drivers at the nexus of the Palearctic and Oriental realms. It provides a foundation for ongoing elucidation of evolutionary relationships and taxonomy. As the first in-depth biogeographic study of Iranian herpetofauna, focused on snakes, it advances regional knowledge while offering opportunities for expanded analysis and comparison across taxa. Overall, these integrated distributions, processes, and patterns provide insights into the origins, diversification pathways, and biodiversity structure underlying Iran’s unique ophiofauna at the crossroads of Asia.

## Supporting information

S1 Fig Similarity dendrogram using Sorenson index. **(**a) Generic Similarity, and (b) Species Similarity. Abbreviations as in Table 1.
(TIF)

S1 Table The distribution data (latitude and longitude) were extracted from multiple sources except those recorded in GBIF are presented below: 1- from previous published literatures and ssbooks [1–58]; 2- occurrence data collected by the authors and their colleagues during numerous fieldworks carried out in recent years; 3- and from the herpetological collections of several Zoological Museums of the following Iranian Universities: Shahid Bahonar University of Kerman; Hakim Sabzevari University of Sabzevar; Razi University of Kermanshah; Shiraz University; Golestan University; Mazandaran University; Tehran University; and Shahid Beheshti University. The Species Diversity Museums of Provincial Departments of Environment (DOE) were also investigated in the present study as follow: Ardebil, Bushehr, East Azarbayjan, West Azarbayjan, Esfahan, Fars, Guilan, Golestan, Hormozgan, Ilam, Kerman, North Khorasan, Khorasan-e-Razavi, Khuzestan, Kohgiluyeh and Boyer-Ahmad, Lorestan, Mazandaran, Semnan, Sistan and Baluchestan, and Yazd. Supplementary distribution data were retrieved from the Zoological Collection of the Department of Venomous Animals and Antiserum Production, Razi Vaccine and Serum Research Institute, Hesarak, Alborz Province.
(DOCX)

S2 Table Biogeographical characteristics of each thirteen physiographic regions of Iran. Climate Types [1], Main Biomes and Ecoregions [2].
(DOCX)

S3 Table The distance matrix of Jaccard similarity index between snake genera assemblages in Iran and surrounding regions for genera. The data are clustered using UPGMA and presented as dendrogram in Fig 4A.
(DOCX)

S4 Table The distance matrix of Jaccard similarity index between snake species and subspecies assemblages in Iran and surrounding regions for genera. The data are clustered using UPGMA and presented as dendrogram in Fig 4B.
(DOCX)
