# Supplementary figures and images for: Biogeography of the Iranian snakes
Source: PLoS One. 2024 Oct 16;19(10):e0309120. doi: 10.1371/journal.pone.0309120 (PMC11482698; doi:10.1371/journal.pone.0309120)

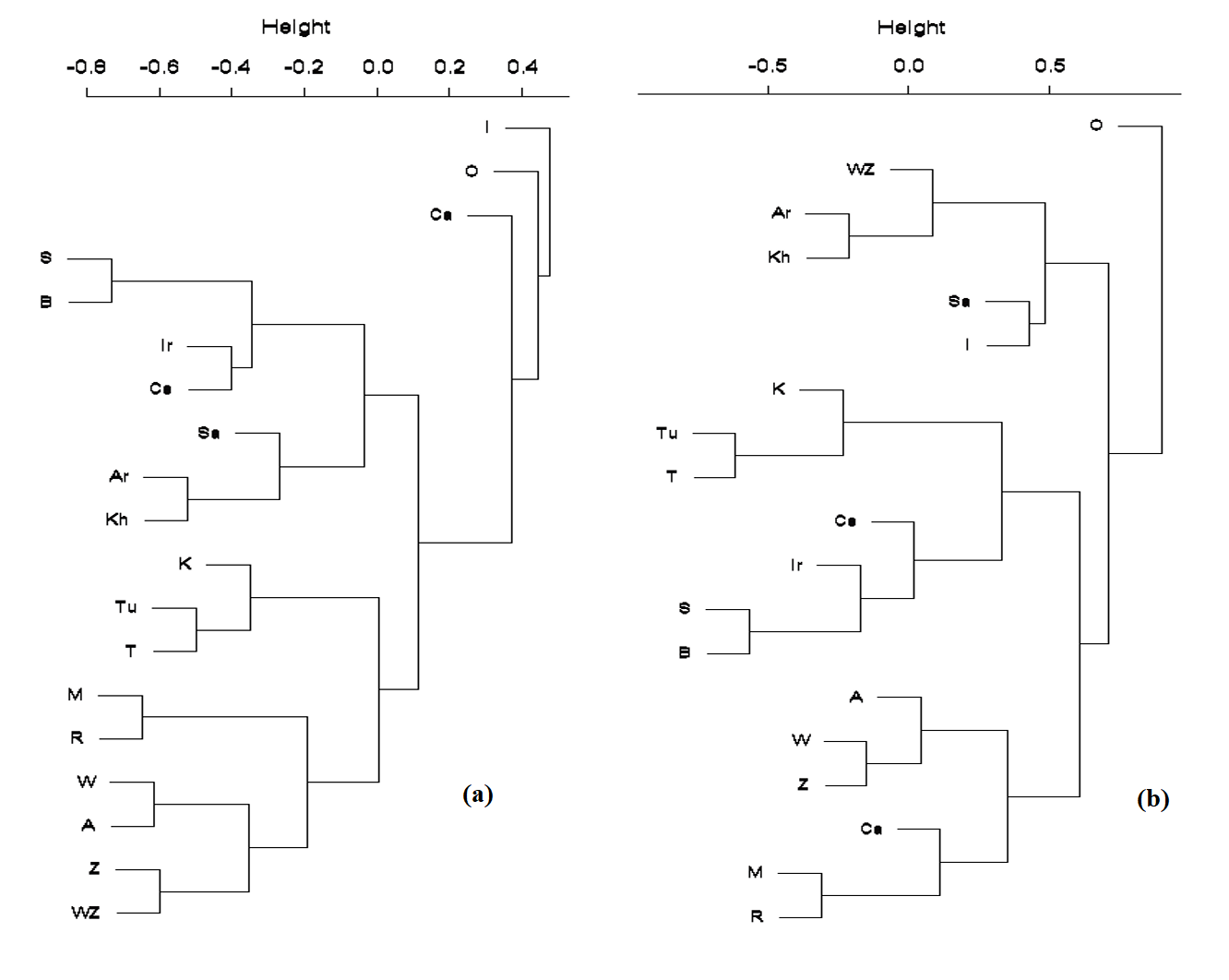

Supplement: S1 Fig — (a) Generic Similarity, and (b) Species Similarity. Abbreviations as in Table 1. (TIF) [file pone.0309120.s001.tif]
