# Supplementary material for: Biogeography of the Iranian snakes
Source: PLoS One. 2024 Oct 16;19(10):e0309120. doi: 10.1371/journal.pone.0309120 (PMC11482698; doi:10.1371/journal.pone.0309120)
Supplement: S1 Table — The Species Diversity Museums of Provincial Departments of Environment (DOE) were also investigated in the present study as follow: Ardebil, Bushehr, East Azarbayjan, West Azarbayjan, Esfahan, Fars, Guilan, Golestan, Hormozgan, Ilam, Kerman, North Khorasan, Khorasan-e-Razavi, Khuzestan, Kohgiluyeh and Boyer-Ahmad, Lorestan, Mazandaran, Semnan, Sistan and Baluchestan, and Yazd. Supplementary distribution data were retrieved from the Zoological Collection of the Department of Venomous Animals and Antiserum Production, Razi Vaccine and Serum Research Institute, Hesarak, Alborz Province. (DOCX) [file pone.0309120.s002.docx]

S1 Table.

| **Leptotyphlopidae Stejneger, 1892** |  |
| --- | --- |
| *Myriopholis blanfordii* (Boulenger, 1890) | 26.136, 61.520; 29.762, 60.638; 29.303, 51.014; 31.950, 50.970; 31.950, 49.081 |
| *Myriopholis hamulirostris* (Nikolsky, 1907) | 30.309, 51.027; 32.387, 48.478 |
| *Myriopholis macrorhyncha* (Jan, 1860) | 26.903, 55.392; 27.080, 52.701; 27.406, 56.255; 27.906, 52.232; 27.916, 53.432; 28.040, 53.263; 28.307, 55.192; 28.359, 54.541; 28.461, 53.067; 28.501, 53.605; 29.165, 53.267; 29.179, 52.438; 29.792, 51.607; 31.771, 49.503; 32.992, 48.192; 33.144, 47.722; 33.358, 46.427; 33.674, 46.564; 33.743, 46.485; 34.004, 50.544; 34.875, 50.149; 35.639, 50.184; 35.690, 50.542 |
| **Typhlopidae Merrem, 1820** |  |
| *Xerotyphlops luristanicus* Torki, 2017 | 34.116, 47.881 |
| *Xerotyphlops vermicularis* (Merrem, 1820) | 28.495, 53.616; 28.815, 56.329; 28.827, 56.273; 29.027, 52.425; 29.149, 56.703; 29.301, 56.884; 29.702, 52.521; 30.198, 57.559; 30.242, 54.055; 30.745, 52.695; 32.734, 47.098; 32.778, 48.264; 33.472, 46.633; 35.216, 52.482; 35.555, 51.762; 35.578, 53.379; 35.824, 51.461; 36.351, 50.015; 36.379, 58.129; 36.388, 52.800; 36.399, 52.429; 36.483, 52.283; 36.560, 53.252; 36.604, 52.548; 36.620, 53.002; 36.697, 53.429; 36.717, 53.833; 36.782, 53.798; 36.799, 54.817; 36.901, 46.228; 36.937, 50.528; 37.243, 48.559; 37.308, 49.405; 37.363, 47.701; 37.65, 51.716; 38.811, 46.193 |
| **Erycidae Pyron, Reynolds and Burbrink, 2014** |  |
| *Eryx elegans* (Gray, 1849) | 36.928, 57.818; 37.455, 59.168; 36.105, 57.795; 36.620, 59.612;  36.937, 59.767; 37.754, 58.162; 32.867, 59.443; 33.718, 59.361;  33.712, 59.241; 33.725, 59.264; 37.128, 57.970; 36.773, 54.465;  37.036, 59.300; 37.805, 57.943; 37.977, 57.328; 37.525, 57.271;  37.549, 58.261; |
| *Eryx jayakari* Boulenger, 1888 | 29.212, 51.160; 30.104, 48.942; 30.201, 50.414; 30.503, 49.667; 31.248, 48.397; 31.252, 49.261; 31.329, 48.691; 31.376, 47.783; 31.487, 48.564; 31.509, 48.559; 31.512, 48.633; 31.684, 48.420; 31.717, 48.184; 31.721, 48.115; 31.907, 49.188; 31.936, 47.868; 32.053, 48.064; 32.319, 48.797; 32.465, 48.321; 32.477, 48.286 |
| *Eryx miliaris* (Pallas, 1773) | 32.950, 60.260; 31.030, 61.489; 36.568, 61.096; 35.789, 60.195; 36.221, 58.780; 36.231, 57.661; 33.536, 59.477; 32.871, 59.240; 35.258, 60.638; 35.726, 50.714; 31.087, 61.793; 34.445, 51.161; 31.158, 61.736; 31.187, 61.780; 35.503, 53.406; 34.109, 51.571; 34.109, 51.571; 35.019, 58.772; 32.871, 59.240; 35.725, 50.366; 35.774, 50.054; 35.376, 60.704; 35.909, 57.123; 35.742, 50.632; 33.608, 59.993; 36.437, 55.099; 34.111, 56.471; 33.947, 56.274; 37.304, 56.011; 36.917, 56.433; 36.094, 58.292; 36.639, 57.067; 35.579, 59.238; 33.726, 59.184; 27.241, 60.389; 37.373, 54.557; 37.899, 55.959; 37.235, 54.721; 35.701, 54.407; 36.178, 54.322; 36.366, 59.726; 35.309, 57.429; 35.725, 50.433; 35.724, 50.709; 34.145, 51.473; 34.310, 51.869 |
| *Eryx sistanensis* Eskandarzadeh, Rastegar-Pouyani, Rastegar-Pouyani, Zargan, Hajinourmohamadi, Nazarov, Sami, Rajabizadeh, Nabizadeh and Navaian, 2020 | 31.035, 61.750; 25.668, 59.290; 27.326, 56.978; 25.933, 61.732; 27.504, 56.852; 30.465, 61.033 |
| *Eryx* sp. Eskandarzadeh | 31.578, 49.699; 31.597, 48.540; 38.074, 46.221; 34.447, 45.894;  33.638, 46.559; 33.125, 46.158; 37.412, 47.660; 36.3, 50.279;  36.276, 49.974; 37.620, 44.808; 38.491, 45.089; 34.757, 50.045;  36.285, 50.181; 36.580, 48.513; 36.648, 48.632; 36.128, 46.525;  32.383, 48.423; 31.243, 49.485; 29.645, 51.673; 29.613, 52.668;  36.032, 46.962; 31.809, 54.181; 36.621, 47.426; 35.88, 50.920;  33.665, 46.755; 34.116, 49.652; 31.558, 48.494; 38.947, 46.632; 36.633, 47.691; 36.841, 50.020; 34.466, 51.171 |
| **Colubridae Oppel, 1811** |  |
| *Boiga trigonata melanocephala* (Annandale 1904) | 27.109, 61.365; 27.246, 56.595; 27.278, 61.366; 27.300, 56.276; 27.830, 58.055; 27.861, 59.307; 27.983, 57.761; 28.050, 57.967; 28.072, 61.342; 28.196, 62.070; 28.583, 57.708; 28.666, 57.716; 28.801, 57.626; 28.806, 60.125; 28.966, 56.591; 29.131, 58.383; 29.253, 58.751; 29.267, 60.339; 30.954, 58.376; 31.111, 61.415; 31.234, 60.991; 31.878, 58.637; 32.792, 58.896; 32.965, 57.028; 32.995, 59.469; 33.525, 56.951; 33.567, 59.630; 34.815, 60.343; 34.863, 60.384; 35.863, 59.615; 36.044, 57.935; 36.301, 59.435 |
| *Coronella austriaca* Laurenti, 1768 | 35.923, 50.444; 35.902, 52.132; 36.025, 53.822; 36.237, 52.910; 35.902, 52.132; 36.160, 51.297; 36.235, 51.302; 36.479, 51.348; 37.732, 48.950; 36.386, 51.418; 36.762, 49.805; 37.065, 49.240 |
| *Dolichophis andreanus* (Werner, 1917) | 29.721, 52.849; 29.666, 52.483; 29.934, 52.883; 33.565, 48.142; 34.116, 47.881; 33.740, 47.524; 33.748, 47.750; 33.181, 47.719; 32.832, 48.628; 33.052, 47.221; 29.065, 57.918; 33.414, 48.984; 33.052, 47.221; 34.064, 49.816; 33.736, 46.620; 30.068, 50.349; 31.817, 50.068; 29.280, 52.983; 33.157, 47.303; 33.351, 46.537; 33.726, 46.500; 34.164, 49.658 |
| *Dolichophis jugularis* (Linnaeus, 1758) | 36.883, 54.349; 36.316, 54.141; 36.420, 53.136; 36.366, 52.665; 36.540, 49.382; 38.620, 44.767; 30.100, 50.917; 33.184, 47.365; 33.726, 46.500; 33.716, 46.513; 34.195, 45.852; 34.944, 46.385; 36.398, 52.919; 29.568, 51.382; 37.274, 58.117 |
| *Dolichophis schmidti* (Nikolsky, 1909) | 36.566, 58.583; 36.681, 54.203; 35.968, 53.514; 36.678, 53.442; 36.099, 52.990; 36.499, 52.997; 36.307, 52.627; 36.367, 52.105; 36.379, 51.782; 36.050, 51.415; 36.503, 51.285; 36.884, 50.294; 37.300, 49.166; 38.499, 44.761; 37.337, 44.921; 39.384, 48.118; 38.513, 47.315; 37.372, 46.445; 36.319, 46.579; 35.523, 48.484; 37.167, 54.304; 37.042, 45.117; 35.507, 46.160; 37.249, 49.956; 36.359, 50.012; 37.347, 55.588 |
| *Eirenis* *collaris* (Méntériés, 1832) | 34.414, 47.443; 34.393, 47.183; 34.280, 47.567; 34.766, 46.534; 34.282, 46.243; 34.158, 46.110; 34.133, 46.478; 33.114, 47.359; 34.745, 48.506; 35.301, 46.954; 37.455, 45.683; 37.449, 46.264; 38.937, 45.625; 34.938, 50.017; 36.001, 51.032; 32.671, 50.946; 33.972, 46.128; 33.830, 46.458; 33.776, 46.159; 33.082, 47.314; 36.632, 47.692; 35.821, 47.061; 36.784, 49.405; 36.843, 50.019; 36.529, 50.183; 36.358, 50.014; 35.663, 50.286; 35.654, 51.066; 35.840, 51.481; 35.745, 51.782; 34.880, 50.120; 33.248, 49.952  32.78764, 48.50502; |
| *Eirenis* *coronella* (Schlegel, 1837) | 31.719, 49.100; 31.719, 48.375; 32.371, 48.287 |
| *Eirenis* *coronelloides* (Jan, 1862) | 34.260, 46.625; 34.069, 46.537; 32.426, 49.122; 33.5325151, 46.1509203 |
| *Eirenis* *kermanensis* Rajabizadeh, Schmidtler, Orlov and Soleimani, 2012 | 28.859, 56.259 |
| *Eirenis* *medus* Chernov, 1940 | 30.068, 57.198; 36.295, 59.388; 36.260, 49.939; 35.441, 49.236; 32.563, 50.423; 35.981, 51.405; 35.833, 51.460; 35.512, 59.783; 36.508, 54.378; 35.727, 52.928; 34.105, 48.973; 35.926, 51.478; 35.922, 51.481 |
| *Eirenis* *modestus* (Martin, 1838) | 39.247, 44.446; 38.701, 46.600; 38.391, 48.577 |
| *Eirenis nigrofasciatus* (Nikolsky, 1907) | 28.747, 54.554; 35.175, 45.984; 34.701, 44.963; 33.115, 46.156;  32.583, 48.399; 29.622, 51.67; 29.467, 53.277; 26.949, 55.582;  28.856, 56.379; 30.059, 54.304; 32.368, 48.635; 36.714, 44.475;  28.698, 56.297 |
| *Eirenis occidentalis* Rajabizadeh, Nagy, Adriaens, Avci, Masroor, Schmidtler, Nazarov, Esmaeili and Christiaens, 2016 | 34.463, 45.879 |
| *Eirenis* *persicus* (Anderson, 1872) | 29.682, 51.828; 33.659, 46.462; 33.071, 47.706; 32.371, 49.044; 29.792, 51.607; 29.702, 52.521; 34.406, 46.061; 34.464, 45.859; 32.368, 48.635; 31.899, 49.298; 31.21, 51.769; 29.622, 51.67; 29.557, 52.491; 28.503, 53.567; 29.203, 54.332; 28.926, 50.857; 33.045, 47.745; 29.658, 52.011 |
| *Eirenis* cf*. persicus* Rajabizadeh | 35.848, 51.011; 35.023, 50.058; 35.705, 51.371 |
| *Eirenis* *punctatolineatus* (Boettger, 1892) | 36.246, 57.761; 34.993, 46.417; 35.161, 54.392; 34.906, 52.134; 34.631, 47.630; 34.864, 51.366; 36.148, 50.782; 36.173, 49.897; 36.121, 49.015; 35.95, 46.6; 37.053, 47.356; 36.956, 45.353; 36.853, 50.019; 35.773, 51.22; 35.918, 51.510; 35.756, 51.830; 35.737, 52.506; 36.375, 54.737; 36.560, 59.588; 35.821, 51.308; 36.119, 45.782 |
| *Eirenis* *punctatolineatus condoni* (Boulenger, 1920) | 28.667, 57.699; 28.582, 57.760; 29.093, 54.575; 29.180, 53.835; 28.908, 53.602; 28.621, 53.130; 33.132, 47.694; 32.690, 49.103; 32.208, 50.123; 32.697, 49.940; 32.834, 51.067; 33.984, 51.163; 34.046, 46.350; 33.972, 46.128; 33.726, 46.500; 33.658, 46.026; 33.400, 51.511; 32.765, 51.066; 29.700, 52.519; 33.232, 48.181; 33.238, 48.183; 28.878, 56.393 |
| *Eirenis rafsanjanicus* Akbarpour, Rastegar-Pouyani, Fathinia and Rastegar-Pouyani, 2020 | 30.362, 55.428 |
| *Eirenis* *rechingeri* Eiselt, 1971 | 31.402, 51.528; 29.868, 51.808 |
| *Eirenis thospitis* Schmidtler and Lanza, 1990 | 37.58, 44.63 |
| *Eirenis* *walteri* (Boettger, 1888) | 36.539, 61.173; 34.55, 60.133; 29.059, 57.902; 27.837, 60.207; 30.277, 57.062; 28.666, 57.758; 25.809, 61.498; |
| *Eirenis yassujicus* Fathinia, Rastegar-Pouyani and Shafaeipour, 2019 | 30.630, 51.419 |
| *Elaphe dione* (Pallas, 1773) | 36.809, 54.320; 36.404, 53.648; 36.695, 53.453; 36.252, 53.047; 36.396, 52.381; 36.872, 53.617; 36.513, 51.284; 36.763, 50.666; 37.014, 50.244; 37.055, 49.901; 37.331, 49.599; 37.118, 49.447; 37.232, 49.151; 37.644, 48.786; 36.204, 53.598; 37.027, 50.014; 37.181, 50.026; 37.306, 49.993; 36.584, 52.171 |
| *Elaphe urartica* Jablonski, Kukushkin, Avcı, Bunyatova, Kumlutaş, Ilgaz, Polyakova, Shiryaev, Tuniyev, Jandzik, 2019 | 38.433, 44.817; 38.629, 48.095; 37.062, 48.835; 37.143, 45.110; 35.252, 47.372; 35.004, 46.263; 36.700, 54.546; 36.183, 53.736; 35.634, 52.634; 39.176, 44.310 |
| *Hemorrhois nummifer* (Reuss, 1834) | 36.106, 51.359; 35.505, 46.238; 37.390, 58.220; 37.442, 57.638; 37.773, 56.660; 38.722, 46.453; 36.521, 47.651; 34.771, 48.332; 33.580, 48.903; 33.516, 47.541; 36.087, 45.432; 37.894, 44.432; 38.962, 47.541; 36.158, 53.615 |
| *Hemorrhois ravergieri* (Ménétriés, 1832) | 37.566, 56.616; 36.833, 55.633; 37.300, 55.083; 37.066, 54.500; 36.933, 54.083; 29.650, 51.566; 28.883, 51.283; 28.883, 52.066; 29.666, 52.583; 28.850, 52.566; 28.516, 52.983; 28.650, 53.300; 28.950, 53.616; 29.150, 54.350; 28.966, 54.983; 27.400, 56.183; 29.300, 55.100; 29.883, 55.766; 28.716, 56.283; 29.466, 56.400; 29.933, 56.550; 30.450, 55.916; 31.533, 55.683; 30.766, 56.633; 30.233, 57.116; 31.333, 55.000; 34.016, 46.166; 33.416, 46.550; 30.883, 51.533; 34.900, 50.083; 35.316, 49.283; 35.316, 48.200; 38.583, 44.600; 38.566, 45.483; 38.383, 47.550; 35.733, 50.166; 36.200, 51.816; 36.183, 51.716; 36.000, 51.983; 35.966, 53.483; 35.966, 53.600; 36.033, 53.550; 36.116, 53.700; 36.050, 53.683; 36.183, 50.283; 36.116, 45.766; 36.116, 45.766; 36.700, 58.550; 33.716, 46.500; 33.700, 46.466; 33.766, 46.483; 36.050, 57.950; 37.019, 59.654; 36.411, 59.301; 36.008, 59.448; 34.456, 51.188; 35.866, 52.724; 37.373, 48.803; 38.885, 46.287; 33.964, 48.659; 33.605, 46.410; 32.578, 51.397; 32.216, 50.795; 29.446, 57.332 |
| *Lycodon bicolor* (Nikolsky, 1903) | 38.135, 56.492; 38.152, 57.173; 37.423, 58.074; 31.290, 61.678; 27.781, 61.832; 36.527, 60.337; 36.191, 57.547; 36.880, 54.317; 36.510, 54.734 |
| *Lytorhynchus gaddi* Nikolsky, 1907 | 29.434, 50.836; 31.077, 48.728; 31.548, 48.496; 32.313, 48.806; 32.380, 48.410 |
| *Lytorhynchus maynardi* Alcock and Finn, 1897 | 26.219, 60.283; 31.133, 61.616; 31.135, 61.767; 27.715, 62.266 |
| *Lytorhynchus ridgewayi* Boulenger, 1887 | 26.756, 62.980; 27.200, 62.574; 29.791, 60.834; 31.157, 60.779; 27.908, 58.216; 27.139, 57.118; 27.283, 56.141; 28.901, 56.539; 29.015, 58.215; 30.200, 55.500; 30.186, 55.474; 29.007, 51.071; 30.359, 52.641; 30.626, 53.706; 31.200, 57.200; 32.699, 58.612; 33.703, 60.150; 36.118, 61.135; 36.903, 58.067; 37.340, 58.143;  37.324, 57.333; 36.000, 56.100; 34.00, 49.700; 34.700, 50.800; 35.100, 51.400; 35.725, 50.435; 31.763, 48.334; 31.314, 48.905 |
| *Oligodon transcaspicus* (Nikolsky, 1902) | 36.527, 60.336; 37.584, 54.903; 37.455, 58.507; 37.551, 57.694; 36.304, 59.485; 37.636, 54.817, 36.310, 60.480, 36.186, 59.508; |
| *Persiophis fahimii* Rajabizadeh, Pyron, Nazarov, Poyarkov, Adriaens and Herrel, 2020 | 28.458, 56.3628 |
| *Platyceps atayevi* (Tuniyev and Shammakov, 1993) | 37.500, 56.850; 37.450, 59.116; 37.100, 58.500; 34.507, 60.586; 33.728, 59.242; 37.497, 58.940; 37.558, 58.501; 38.036, 56.457; 37.567, 56.732; 37.323, 54.524 |
| *Platyceps karelini* (Brandt, 1838) | 25.316, 60.616; 33.866, 59.783; 30.283, 57.083; 29.500, 60.866; 33.844, 50.827; 33.898, 51.436; 34.709, 51.389; 33.023, 52.482; 32.963, 53.411; 33.053, 54.257; 36.041, 55.363; 36.743, 59.698; 36.131, 58.974; 35.563, 59.563; 36.069, 60.179; 36.476, 61.018; 32.533, 60.342; 31.440, 60.439; 31.198, 61.362; 30.472, 61.297; 35.532, 61.199; 33.578, 59.079; 33.498, 59.984; 32.564, 59.391; 35.055, 51.422 |
| *Platyceps karelini* *chesneii* (Martin 1838) | 31.833, 48.416; 30.283, 57.083; 30.433, 49.100; 31.545, 48.305; 29.984, 48.577; 30.616, 49.132; 28.982, 50.98; 29.466, 51.083; 29.492, 51.752; 31.644, 52.242; 34.389, 45.657; 29.532, 52.523; 29.266, 51.200; 28.966, 50.833; 34.400, 45.483; 29.600, 52.533; 31.566, 48.183; 30.238, 49.706; 32.389, 48.421 |
| *Platyceps mintonorum* (Mertens, 1969) | 31.033, 61.500; 29.500, 60.866; 30.864, 61.28; 29.485, 60.621;  28.027, 62.525 |
| *Platyceps najadum* (Eichwald, 1831) | 38.466, 44.613; 36.416, 54.966; 37.340, 55.622; 36.929, 55.589; 36.797, 54.381; 36.805, 53.266; 36.687, 53.502; 36.673, 52.963; 36.607, 53.047; 36.565, 53.146; 36.406, 53.143; 36.247, 53.444; 36.131, 53.617; 36.501, 52.778; 36.067, 52.569; 35.934, 52.075; 35.878, 51.551; 36.781, 50.886; 36.645, 50.760; 37.368, 48.991; 36.649, 49.101; 35.208, 49.299; 34.105, 49.870; 34.649, 48.574; 34.884, 47.431; 35.118, 46.376; 36.349, 45.585; 36.825, 46.464; 37.176, 47.563; 37.873, 46.882; 37.699, 47.937; 38.769, 46.333; 39.077, 44.421; 38.254, 44.663; 33.000, 47.416; 37.450, 59.100; 33.133, 47.383; 33.833, 46.316; 33.750, 46.533; 37.450, 58.633; 33.133, 47.100; 37.100, 58.516; 37.733, 44.782 |
| *Platyceps najadum albitemporalis* (Darevsky and Orlov, 1994) | 38.357, 48.771; 38.340, 48.376 |
| *Platyceps rhodorachis* (Jan in De Filippi, 1865) | 33.933, 46.133; 32.700, 46.466; 32.933, 47.350; 33.800, 46.566; 26.533, 59.950; 30.416, 57.700; 28.966, 50.833; 28.650, 59.016; 27.516, 62.750; 29.600, 52.533; 35.216, 60.433; 31.283, 49.600; 26.650, 55.883; 30.283, 57.083; 31.933, 49.300; 37.366, 48.233; 31.683, 49.416; 31.983, 49.316; 31.533, 49.866; 26.150, 61.450; 27.200, 60.700; 29.550, 51.766; 31.016, 49.433; 28.500, 53.566; 27.266, 54.333; 33.033, 47.466; 33.600, 48.150; 34.116, 47.883; 34.066, 47.966; 32.383, 48.650; 31.316, 48.683; 35.833, 51.366; 36.216, 57.533; 28.833, 57.900; 30.333, 50.766; 26.833, 57.250; 35.216, 60.350; 36.216, 57.683; 27.866, 60.183; 32.600, 58.300; 34.366, 45.716; 36.300, 60.433; 35.616, 48.183; 29.500, 60.866; 25.300, 60.633; 27.316, 56.283; 26.833, 57.416; 36.266, 60.650; 36.400, 55.683; 29.500, 60.866; 28.750, 54.550; 33.750, 45.850; 34.716, 47.950; 32.866, 59.216; 27.166, 60.633; 33.116, 54.750; 32.500, 55.583; 36.466, 54.616; 27.683, 60.283; 27.500, 60.366; 28.500, 60.666; 34.566, 60.566; 32.533, 60.083; 26.233, 61.400; 25.316, 60.616; 36.884, 59.025; 36.403, 59.310; 33.493, 51.763; 32.756, 51.322; 33.317, 47.042; 30.207, 51.896; 27.692, 61.423; 27.789, 61.944; 32.394, 48.464; 32.442, 48.148 |
| *Platyceps* cf*. r. rhodorachis* Schätti, 2014 | 36.130, 46.317; 35.890, 46.766; 35.271, 46.210 |
| *Platyceps schmidtleri* (Schätti and McCarthy, 2001) | 29.200, 56.600; 30.666, 51.600; 31.316, 48.683; 34.116, 47.883; 29.233, 56.600; 29.666, 51.983; 31.516, 51.166; 31.716, 50.283; 30.883, 52.683; 33.033, 50.650; 33.750, 47.616; 28.500, 53.566; 30.283, 57.083; 33.166, 47.666; 29.000, 52.483; 32.050, 51.466; 29.600, 52.533; 29.600, 52.533; 30.750, 50.750; 32.866, 48.733; 30.666, 51.583; 30.716, 51.633; 33.979, 46.215; 34.379, 47.709; 33.026, 47.313; 31.540, 48.412; 33.158, 48.166 |
| *Platyceps ventromaculatus* (Gray, 1834) | 28.233, 61.250; 27.500, 60.366; 25.363, 60.814; 26.223, 61.685; 25.284, 61.609 |
| *Rhynchocalamus levitoni* (Torki, 2017) | 33.640, 46.038; 32.952, 47.344; 33.033, 47.300 |
| *Rhynchocalamus satunini* (Nikolsky, 1899) | 34.584, 49.891; 35.191, 51.730; 29.464, 53.142; 32.403, 48.528; 32.069, 49.011; 34.166, 46.682; 36.919, 45.012; 38.758, 46.023 |
| *Spalerosophis diadema* *cliffordii* (Schlegel, 1837) | 30.563, 50.116; 29.285, 51.123; 28.873, 51.362; 27.809, 52.281; 27.257, 52.950; 27.417, 53.171; 27.372, 56.227; 27.204, 57.066; 26.491, 57.205; 25.777, 57.791; 28.438, 56.209; 28.335, 55.893;  27.976, 57.873; 28.428, 57.809; 28.618, 59.165; 29.128, 58.449; 31.525, 48.524; 30.238, 49.706; 32.394, 48.464 |
| *Spalerosophis schirasianus* (Jan, 1863) | 29.050, 55.085; 31.158, 53.587; 30.419, 57.682; 36.327, 59.457; 36.799, 55.767; 35.224, 52.527; 36.031, 49.716; 34.737, 51.004; 34.381, 50.887; 26.596, 61.219; 28.167, 57.469; 35.872, 57.226; 35.055, 51.422; 35.721, 50.368; 34.444, 51.221; 34.299, 51.944; 35.721, 50.368; 35.111, 51.770; 30.413, 57.685; 28.674, 57.811; 28.408, 56.378 |
| *Spalerosophis microlepis* Jan, 1865 | 33.383, 46.083; 33.433, 46.333; 33.733, 46.000; 33.716, 46.483; 28.933, 56.983; 28.750, 56.600; 28.733, 56.500; 29.702, 52.514; 29.666, 52.746; 34.623, 50.784; 34.379, 51.093; 34.885, 51.049; 34.957, 50.302; 34.270, 50.346; 34.921, 48.852; 34.560, 48.236; 33.137, 46.347; 32.657, 47.182; 33.797, 47.006; 31.914, 49.555; 32.063, 51.225 |
| *Telescopus fallax* *iberus* (Eichwald, 1831) | 36.083, 53.683; 36.083, 53.383; 36.183, 53.166; 36.066, 53.016; 36.000, 52.783; 35.950, 52.300; 35.900, 52.033; 36.066, 51.883; 38.451, 48.850; 36.626, 47.654; 37.192, 50.042; 36.893, 49.984; 35.724, 50.433; 35.654, 50.526; 35.770, 51.217; 35.831, 51.461; 36.406, 52.812; 36.711, 53.816; 36.779, 54.463; 36.533, 53.131; 35.796, 51.238; 36.081, 53.031; 37.685, 47.761; 37.563, 45.147; 34.635, 46.597; 34.508, 47.432; 34.833, 47.783; 32.900, 50.112 |
| *Telescopus nigriceps* Ahl, 1924 | 35.265, 46.223; 34.996, 46.157; 34.272, 46.509; 33.927, 48.860 |
| *Telescopus rhinopoma* (Blanford, 1874) | 32.516, 60.083; 32.200, 59.316; 29.116, 60.166; 29.250, 57.916; 29.383, 57.166; 28.833, 56.733; 30.400, 57.750; 30.350, 56.783; 29.716, 56.100; 30.766, 55.566; 29.800, 54.883; 28.733, 55.083; 30.433, 54.216; 29.950, 53.850; 28.650, 54.033; 28.766, 53.066; 29.400, 52.350; 28.716, 51.516; 29.400, 51.183; 30.916, 51.316; 31.733, 49.533; 35.450, 52.483; 35.350, 51.300; 36.116, 54.433; 36.000, 59.416; 35.262, 54.428; 29.702, 52.512; 30.932, 51.322; 33.936, 50.596; 29.489, 60.923; 32.835, 59.341; 36.131, 59.868; 36.520, 54.902 |
| *Telescopus tessellatus tessellatus*(Wall, 1908) | 35.324, 46.650; 34.713, 46.650; 33.292, 47.792; 33.402, 48.539; 32.850, 51.967; 34.713, 50.737; 33.987, 50.561; 34.096, 49.902; 35.727, 51.681; 35.923, 50.890 |
| *Telescopus tessellatus martini* Schmidt, 1939 | 28.991, 52.881; 31.944, 48.794; 32.354, 48.311; 31.781, 49.514; 33.108, 46.342; 34.495, 45.815 |
| *Zamenis hohenackeri* (Strauch, 1873) | 39.317, 44.301; 37.527, 47.949; 32.324, 50.256; 38.779, 46.510; 38.320, 46.239; 38.389, 48.527 |
| *Zamenis longissimus* (Laurenti, 1768) | 36.016, 53.666; 39.354, 44.514; 36.761, 50.859; 38.467, 44.371; 36.583, 47.025; 34.055, 47.881 |
| *Zamenis persicus* (F. Werner, 1913) | 36.683, 54.650; 36.483, 54.050; 36.266, 54.083; 36.050, 53.483; 36.283, 53.466; 36.533, 53.150; 36.533, 52.633; 37.249, 49.952; 37.188, 50.196; 36.717, 53.720; 36.303, 52.870; 36.435, 57.294; 37.429, 48.415; 38.857, 47.002 |
| **Natricidae Bonaparte, 1838** |  |
| *Natrix natrix scutata* (Pallas, 1771) | 36.550, 53.183; 36.683, 53.066; 36.550, 52.733; 36.550, 52.283; 36.550, 51.983; 36.650, 51.450; 36.800, 50.833; 36.883, 50.666; 37.200, 50.233; 37.433, 49.450; 36.964, 49.565; 36.088, 53.047; 36.964, 55.03 |
| *Natrix tessellata* (Laurenti, 1768) | 36.833, 54.666; 36.733, 53.783; 36.850, 53.633; 36.716, 53.533; 36.166, 53.616; 36.150, 53.550; 36.133, 53.283; 36.666, 53.316; 36.783, 53.150; 36.650, 52.416; 36.683, 52.633; 36.666, 52.866; 36.633, 53.050; 36.500, 52.850; 36.550, 52.616; 36.500, 52.350; 37.583, 48.583; 37.966, 47.516; 38.533, 44.833; 37.250, 44.883; 36.916, 45.366; 36.016, 45.933; 35.866, 46.983; 33.466, 46.400; 30.550, 48.666; 29.500, 51.833; 29.483, 52.683; 30.683, 52.150; 30.333, 52.216; 33.716, 46.500; 33.550, 46.833; 33.116, 47.350; 33.216, 46.233; 38.974, 45.516; 37.549, 45.078; 36.653, 47.683; 35.526, 46.154; 33.864, 48.285; 33.713, 48.838; 32.778, 48.263; 34.586, 50.803; 32.664, 51.190; 29.018, 52.431; 36.982, 58.128; 37.492, 58.464 |
| **Psammophiidae Bourgeois, 1968** |  |
| *Malpolon insignitus* *fuscus* (Fleischmann, 1831) | 36.166, 58.650; 36.333, 59.500; 38.016, 56.966; 36.716, 55.616; 36.450, 54.933; 36.200, 54.300; 35.200, 52.483; 35.733, 50.250; 36.450, 48.450; 37.733, 47.950; 36.766, 47.516; 37.800, 46.433; 36.700, 46.716; 36.750, 45.300; 35.716, 47.150; 35.450, 48.083; 34.950, 48.783; 35.133, 46.950; 34.100, 47.100; 33.883, 46.150; 34.016, 48.350; 34.016, 49.583; 27.150, 55.816; 32.566, 47.516; 31.733, 49.500; 30.950, 49.800; 33.283, 50.650; 32.633, 51.816; 35.050, 51.750; 33.716, 46.483; 33.050, 47.316; 33.650, 46.550; 38.974, 45.516; 34.603, 48.463; 34.372, 50.873; 35.600, 50.543; 37.251, 48.569 |
| *Psammophis lineolatus* (Brandt, 1838) | 36.494, 60.996; 36.034, 59.634; 36.069, 57.524; 36.247, 54.668; 36.671, 54.360; 36.987, 54.316; 35.820, 50.932; 34.925, 52.295; 33.581, 57.700; 32.808, 59.194; 31.056, 61.523; 29.538, 60.776; 28.539, 57.876; 30.451, 56.953; 29.614, 52.119; 30.980, 52.690; 31.469, 48.471; 31.880, 48.603; 32.475, 51.811; 33.361, 48.252; 33.397, 50.449; 33.982, 50.625 |
| *Psammophis schokari* (Forskål, 1775) | 36.716, 55.633; 36.633, 55.083; 36.333, 54.950; 36.216, 54.316; 34.800, 50.866; 35.166, 52.433; 34.700, 52.350; 35.200, 54.533; 34.666, 54.583; 34.100, 54.450; 33.783, 55.033; 33.566, 56.833; 32.783, 56.966; 36.366, 59.483; 36.150, 58.516; 35.183, 59.350; 32.300, 49.116; 31.816, 49.816; 29.283, 51.216; 28.900, 51.266; 27.800, 52.266; 28.900, 52.116; 29.683, 52.766; 28.883, 52.600; 28.500, 53.000; 28.650, 53.300; 27.600, 54.416; 27.466, 55.433; 27.383, 56.216; 27.116, 57.000; 26.500, 57.150; 28.550, 56.150; 28.550, 58.150; 28.733, 57.750; 29.100, 58.450; 29.450, 57.200; 30.083, 56.650; 30.466, 55.800; 30.816, 56.500; 29.766, 53.916; 30.133, 55.050; 31.416, 56.250;31.483, 55.733; 31.433, 55.000; 31.566, 54.166; 32.133, 54.666; 32.316, 54.083; 28.666, 61.183; 29.616, 51.650; 35.766, 56.000; 33.683, 45.866; 33.633, 45.966; 32.333, 47.683 |
| *Rhagerhis moilensis* (Reuss, 1834) | 27.166, 56.133; 31.066, 49.233; 32.300, 47.600; 32.400, 47.616; 32.666, 47.300; 30.250, 49.751 |
| **Elapidae Boie, 1827** |  |
| *Bungarus persicus* Abtin, Nilson, Mobaraki, Hosseini and Dehgannejhad, 2014 | 26.747, 62.664; 26.75, 57.841; 26.652, 61.253 |
| *Naja* *oxiana* (Eichwald, 1831) | 29.815, 60.600; 31.637, 60.249; 32.132, 60.194; 33.590, 59.007; 34.572, 60.062; 35.015, 59.886; 36.441, 60.567; 36.202, 59.106; 36.741, 59.787; 35.418, 57.953; 36.326, 57.348; 37.093, 58.227; 37.512, 58.216; 37.591, 57.535; 37.469, 57.041; 37.433, 56.338; 37.486, 55.893; 37.377, 55.525; 37.346, 55.003; 37.106, 54.591; 36.847, 54.470; 36.807, 54.196; 36.679, 54.262; 37.246, 54.157 |
| *Walterinnesia morgani* (Mocquard, 1905) | 32.07, 48.87; 34.458, 45.855; 33.128, 46.120; 31.960, 49.281; 28.901, 50.833; 27.388, 56.759; 33.997, 45.480; 29.635, 51.641; 33.123, 46.166; 27.474, 53.052; 27.466, 53.060; 27.47, 53.059; 27.513, 52.929; 28.359, 54.541; 32.614, 48.507; 33.151, 47.721; 27.517, 57.945; 28.600, 56.133; 28.633, 56.100; 28.400, 58.666; 28.000.0, 57.516; 28.750, 51.466; 27.083, 55.900; 28.616, 56.283 |
| **Viperidae Oppel, 1811** |  |
| *Gloydius caucasicus* (Clade i)  = Kopet Dagh-Eastern Alborz | 37.087, 59.502; 37.000, 59.557; 37.122, 59.326; 37.149, 58.304; 37.489, 57.162; 37.428, 55.865; 37.387, 55.667; 37.107, 55.304; 37.125, 54.766; 36.777, 54.433; 37.373, 56.087; 36.781, 54.952; 36.618, 54.661; 36.541, 54.462; 36.428, 54.390 |
| *Gloydius caucasicus* (Clade ii)  = Lar National Park-Central Alborz | 36.333, 54.050; 36.566, 53.450; 36.566, 53.450; 36.350, 52.300; 36.677, 53.496; 36.690, 53.386; 36.620, 53.342; 36.435, 53.112; 36.399, 53.364; 36.222, 53.474; 36.133, 53.232; 36.417, 52.914; 36.346, 52.705; 36.240, 52.881; 36.036, 52.947; 36.302, 52.562; 36.373, 52.310; 36.149, 52.461; 36.125, 52.364; 35.971, 52.043; 36.364, 52.104; 35.943, 51.870 |
| *Gloydius caucasicus* (Clade iii)  = Central Alborz | 36.609, 51.453; 36.516, 51.378; 36.395, 51.230; 36.459, 51.118; 36.602, 50.936; 36.673, 50.969; 36.675, 50.821; 36.713, 50.670; 36.840, 50.538; 36.679, 50.563; 36.359, 50.895; 36.255, 50.689; 36.109, 50.870; 36.538, 50.469; 36.851, 50.288; 36.983, 50.472; 36.812, 50.126; 36.076, 51.252 |
| *Gloydius caucasicus* (Clade iv)  = Western Alborz-Azerbaijan | 37.133, 49.350; 36.133, 49.300; 36.970, 49.511; 36.860, 49.560; 37.141, 49.198; 37.384, 49.053; 37.382, 48.858; 37.618, 48.973; 37.626, 48.632; 37.426, 48.594; 37.917, 48.616; 37.644, 48.748; 38.410, 48.056; 38.281, 47.885; 38.771, 47.830; 38.865, 47.368; 37.039, 50.000 |
| *Cerastes gasperettii* Leviton and Anderson, 1967 | 32.050, 48.100; 32.050, 48.083; 32.050, 48.033; 32.050, 48.016; 31.950000, 47.816; 32.083, 47.800; 32.116, 47.816 |
| *Echis carinatus sochureki* Stemmler, 1969 | 34.200, 51.662; 35.224, 51.807; 35.181, 52.196; 35.380, 53.117; 35.502, 53.201; 35.937, 53.603; 36.065, 53.720; 32.975, 59.250; 33.231, 60.424; 32.578, 58.665; 30.754, 61.263; 30.928, 60.534; 29.431, 61.083; 28.145, 61.979; 26.963, 62.966; 27.019, 62.443; 27.779, 58.600; 27.166, 57.461; 29.156, 58.460; 28.843, 58.609; 28.643, 57.766; 27.989, 57.777; 27.193, 57.084; 30.422, 57.686; 31.358, 56.864; 31.414, 56.245; 31.886, 56.033; 31.482, 55.736; 28.916, 56.468; 28.770, 56.439; 28.766, 56.357; 28.727, 56.268; 28.681, 56.353; 28.630, 56.233; 28.537, 56.179; 28.464, 56.356; 27.204, 56.287; 27.446, 55.442; 26.551, 54.841; 26.729, 54.263; 27.154, 54.385; 27.352, 53.179; 27.541, 52.892; 28.472, 53.006; 28.594, 53.367; 28.962, 28.962; 29.892, 52.792; 31.160, 53.286; 30.135, 51.524; 29.607, 51.681; 28.890, 51.3; 29.601, 50.558; 30.070, 50.168; 30.607, 50.220; 30.728, 49.725; 31.420, 49.548; 31.700, 49.584; 32.062, 48.837; 31.954, 49.309; 31.850, 49.890; 27.117, 61.683; 26.077, 61.467; 25.745, 58.393; 25.807, 57.422; 27.097, 59.084; 27.037, 60.886; 27.188, 60.626; 33.567, 56.851; 32.041, 54.619; 32.257, 54.090; 33.754, 55.115; 34.038, 54.424; 35.047, 54.604; 35.467, 55.829; 35.605, 55.988; 35.066, 59.117; 36.111, 58.690; 36.235, 57.183; 36.745, 55.826; 36.620, 55.657; 36.330, 54.991; 36.095, 54.396 |
| *Eristicophis macmahoni* Alcock and Finn, 1897 | 30.409, 61.172 |
| *Macrovipera lebetina chernovi* (Chikin and Szczerbak, 1992) | 37.364, 58.749; 37.373, 58.756; 37.575, 58.683; 37.996, 56.550; 37.434, 56.793; 36.853, 55.413; 36.761, 55.434; 36.322, 53.888 |
| *Macrovipera lebetina obtusa* (Dwigubskij, 1832) | 38.974, 45.516; 38.374, 45.070; 38.466, 44.613; 33.155, 50.392; 34.127, 50.237; 34.228, 46.671; 35.167, 46.672; 36.659, 47.274; 36.825, 49.375; 35.663, 51.730; 36.321, 54.273; 36.748, 54.554; 37.155, 55.420; 37.451, 56.478; 37.386, 55.895; 36.853, 55.413; 36.027, 53.602; 38.621, 46.716; 36.235, 53.453; 36.322, 53.888; 34.879, 50.100; 37.467, 45.516; 37.565, 44.912; 35.506, 46.348; 32.682, 50.755 |
| *Macrovipera razii* Oraie, Rastegar-Pouyani, Khosravani, Moradi, Akbari, Sehhatisabet, Shafiei, Stümpel and Joger, 2018 | 28.718, 56.353; 28.852, 56.473; 28.802, 56.524; 29.521, 56.179; 29.958, 55.783; 29.286, 55.155; 29.859, 53.772; 29.712, 52.779; 29.705, 52.753; 28.779, 51.434; 28.724, 51.500; 27.551, 52.968; 27.317, 53.152; 28.691, 53.362; 28.622, 58.298; 30.945, 51.399; 30.775, 50.897; 31.702, 50.109; 31.371, 49.618; 31.751, 49.560; 32.464, 49.583; 32.123, 49.019; 32.604, 48.511; 32.943, 47.215; 33.439, 49.740; 31.559, 55.703; 32.095, 54.711; 28.622, 58.298 |
| *Montivipera kuhrangica* Rajabizadeh, Nilson and Kami, 2011 | 33.337, 49.395; 32.804, 49.834; 30.536, 51.691 |
| *Montivipera latifii* (Mertens, Darevsky and Klemmer, 1967) | 35.956, 52.086; 35.929, 51.773; 35.947, 51.328 |
| *Montivipera raddei* (Boettger, 1890) | 34.717, 48.219; 35.005, 47.428; 35.829, 46.505; 36.644, 47.296; 37.660, 46.198; 38.180, 47.472; 38.559, 44.484; 39.345, 44.264; 36.620, 47.307 |
| *Montivipera raddei albicornuta* (Nilson and Andrén, 1985) | 36.790, 48.516; 36.622, 48.922 |
| *Montivipera wagneri* Nilson and Andrén, 1984 | 37.534, 44.638; 39.324, 44.176 |
| *Pseudocerastes persicus* (Duméril, Bibron, and Duméril, 1854) | 31.733, 49.533; 31.766, 49.500; 27.300, 52.700; 27.300, 52.700; 33.650, 45.983; 28.716, 51.516; 29.816, 60.116; 35.200, 57.133; 34.333, 52.216; 33.250, 52.600; 29.050, 57.700; 29.866, 55.366; 34.734, 50.064; 35.338, 52.318; 36.216, 54.407; 37.372, 57.405; 35.292, 58.178; 35.177, 59.312; 32.591, 59.469; 32.803, 56.797; 31.367, 56.347; 29.288, 60.125; 28.841, 60.173; 27.811, 57.577; 27.460, 55.322; 27.631, 54.195; 28.748, 56.354; 28.595, 56.235; 26.749, 54.258; 27.362, 53.187; 28.649, 51.432; 27.378, 52.634; 29.308, 51.327; 30.085, 51.440; 28.891, 51.378; 30.673, 50.310; 30.815, 50.566; 31.422 49.546; 31.701, 49.593; 31.882, 49.908; 31.929, 49.324; 32.049, 48.900; 27.384, 56.196; 25.326, 60.671;  32.216, 57.442; 30.436, 57.327; 29.927, 55.088; 31.684, 54.285; 32.595, 51.649; 33.747, 46.323; 34.764, 52.177; 35.266, 52.341 |
| *Pseudocerastes urarachnoides* Bostanchi, Anderson, Kami, and Papenfuss, 2006 | 33.383, 46.066; 32.883, 47.266; 33.116, 46.900; 33.666, 46.000 |
| *Vipera* *eriwanensis* (Reuss, 1933) | 38.752, 46.176; 38.236, 47.802 |
| *Vipera eriwanensis ebneri* Knoepffler and Sochurek, 1955 | 37.764, 46.286; 36.591, 46.945; 37.747, 47.571; 37.729, 48.098; 36.035, 51.095; 36.374, 51.007; 35.959, 52.051; 36.248, 51.610; 35.868, 52.689; 37.685, 48.714; 36.394, 51.564 |

References

1. Afroosheh M, Rastegar-Pouyani N, Ghoreishi SK, Kami HG. Comparison of geographic variations in *Typhlops vermicularis* (Merrem, 1820) (Ophidia: Typhlopidae) from the Iranian plateau with Turkey and Turkmenistan. Turk J Zool [Internet]. 2013;37(6):685–92. Available from: https://journals.tubitak.gov.tr/zoology/vol37/iss6/3

2. Afroosheh M, Rastegar-Pouyani N, Kami H. Comments on the distribution and external morphology of *Typhlops vermıcularıs* (Ophıdıa: Typhlopıdae) in Iran. Hamadryad. 2012;36(1):12–6.

3. Aghaie Z, Gharzi A, Rastegar-Pouyani N. Reptile fauna of Doroud, Lorestan Province. In: 16th National and 4th International Conference of Biology. Mashhad, Iran: Ferdowsi University; 2010.

4. Ahmadi L, Kami H, Kavyanifard A. The first record of three species of snake in Lorestan Province (Kuhdasht City). In: 17th National and 5th International Conference of Biology. Kerman, Iran: Shahid Bahonar University; 2012.

5. Akbari H, Jalalpour M, Hojati V, Golmohammadi M. The study of Reptiles and Birds fauna of Abbas Abad Wild Life Refuge in Naein, Isfahan Province. J Anim Biol [Internet]. 2011 Jan 21;3(2):1–12. Available from: https://sanad.iau.ir/Journal/ascij/Article/1090552

6. Asadi A, Montgelard C, Nazarizadeh M, Moghaddasi A, Fatemizadeh F, Simonov E, et al. Evolutionary history and postglacial colonization of an Asian pit viper (*Gloydius halys caucasicus*) into Transcaucasia revealed by phylogenetic and phylogeographic analyses. Sci Rep. 2019 Feb 4;9(1).

7. Asadi A, Salmanian A, Kaboli M. *Eirenis thospitis* Schmidtler et Lanza, 1990 (Reptilia: Colubridae): New to Iran. Russ J Herpetol [Internet]. 2020 Nov 21 [cited 2024 Jul 18];27(6):369–72. Available from: https://doi.org/10.30906/1026-2296-2020-27-6-369-372

8. Chefaoui RM, Hosseinzadeh MS, Mashayekhi M, Safaei-Mahroo B, Kazemi SM. Identifying suitable habitats and current conservation status of a rare and elusive reptile in Iran. Amphib Reptil [Internet]. 2018 [cited 2021 Sep 16];39(3):355–62. Available from: https://brill.com/view/journals/amre/39/3/article-p355_9.xml

9. Eskandarzadeh N, Rastegar-Pouyani N, Rastegar-Pouyani E, Todehdehghan F, Rajabizadeh M, Zarrintab M, et al. Revised classification of the genus *Eryx* Daudin, 1803 (Serpentes: Erycidae) in Iran and neighbouring areas, based on mtDNA sequences and morphological data. Herpetol J. 2020 Jan 1;30(1):2–12.

10. Fathinia B, Feili N, Rastegar-Pouyani N, Gharzi A. Additional specimens of *Eirenis coronelloides* (Jan, 1862) (Ophidia: Colubridae) in western Iran. IJAB [Internet]. 2018 Apr 1;14(2). Available from: https://ijab.um.ac.ir/article_30040_1063d7065060101c281f83a831118a48.pdf

11. Fathnia B, Rastegar Pouyani N, Darvishnia H, Rajabzadeh M. The snake fauna of Ilam Province, southwestern Iran. IJAB [Internet]. 2010 Jul 1;6(1):9–23. Available from: https://ijab.um.ac.ir/article_25252_ddb68de98a6638acc28c7e312d74584c.pdf

12. Feili N, Gharzi A. The Snakes’ fauna of Mehran in Ilam Province. In: 17th National and 5th International Conference of Biology. Kerman, Iran: Shahid Bahonar University; 2012.

13. Ghaffari H, Naghibzadeh A, Salehi F, Miri A, Maleki N. Preliminary study of herpetofauna of Bijar protected area, Kurdistan province, Iran. In: 3rd Iranian Conference on Natural Resources Researches with the emphasis on the Environment. Kurdistan, Iran: University of Kurdistan; 2014.

14. Ghazikhanloo E, Kami H. Morphometric and meristic study of Dione’s snake (*Elaphe dione*) in the Golestan and Mazandaran provinces. In: 17th National and 5th International Conference of Biology. Kerman, Iran: Shahid Bahonar University; 2012.

15. Gholamifard A, Rastegar-Pouyani N. Preliminary study of the herpetofauna of Lamerd and Mohr Townships in southwest of Fars Province, southern Iran. In: 17th National and 5th International Conference of Biology. Kerman, Iran: Shahid Bahonar University; 2012.

16. Gholamifard A, Şahin MK. Range dynamics of *Walterinnesia morgani* (Mocquard, 1905) (Serpentes, Elapidae) throughout climatic oscillations in Iran. Herpetozoa. 2023 Jun 21;36:317–24.

17. Golzarianpour K, Shaykhi J. Preliminary examination of the snake fauna of Golestan province (Gonbad kavous region). In: 17th National and 5th International Conference of Biology. Kerman, Iran: Shahid Bahonar University; 2012.

18. Hasanzadeh N, Shajiee H, Shiravi A. The Study of Snakes Fauna in Tabas County in Yazd Province. J Anim Biol [Internet]. 2013;5(4):33–7. Available from: https://sanad.iau.ir/en/Journal/ascij/DownloadFile/1090510

19. Hojati V, Deymekar M. The study of the snake fauna of Taloo and Shirband hunting prohibited area in Semnan Province, Iran. NBR [Internet]. 2020 Nov 10 [cited 2024 Jul 20];7(3):285–94. Available from: http://nbr.khu.ac.ir/article-1-2922-en.html

20. Hojati V, Faghiri A, Babaei Savasari R. The Study of Amphibians and Reptiles Fauna in Kiasar National Park in Mazandaran Province. J Anim Biol. 2012;4(2):33–44.

21. Hojati V, Kami H, Faghiri A, Ahmadzadeh F. The snakes fauna of Damghan. Environ Sci. 2005 Jan;2(6):1–13.

22. Hojati V, Moghaddas D, Faghiri A. Identification of amphibians and reptiles in Shahid Zare National Park, Sari. J Anim Biol. 2009;1(3):31–8.

23. Hosseinian Yousefkhani S, Rastegar-Pouyani E. Reptiles’ fauna of the Qom province. In: 16th National and 4th International Conference of Biology. Mashhad, Iran: Ferdowsi University; 2010.

24. Hosseinzadeh MS, Ghezellou P, Kazemi SM. Predicting the potential distribution of the endemic snake *Spalerosophis microlepis* (Serpentes: Colubridae), in the Zagros Mountains, western Iran. Salamandra. 2017 May 15;53(2):294-8.

25. Kazemi E, Nazarizadeh M, Fatemizadeh F, Khani A, Kaboli M. The phylogeny, phylogeography, and diversification history of the westernmost Asian cobra (Serpentes: Elapidae: *Naja oxiana*) in the Trans‐Caspian region. Ecol Evol. 2020 Dec 22;11(5):2024–39.

26. Kazemi S, Rajabizadeh M. A report on snake fauna of western part of Qom Province, Iran. In: 2nd National Conference of Animal Science. Rasht, Iran: Guilan University; 2007. p. 184–5.

27. Khormizi MZ, Safaei-Mahroo B, Najafabadi MJ, Salemi A, Dehnavi HD, Meybodi MN, Alian MM, Ghaffari H. Diversity and distribution of snake fauna (Squamata: Serpentes) in Yazd Province, Iran. Herpetol Notes. 2021 Dec 19;14:1449-62.

28. Lee JL, Yushchenko PV, Milto KD, Rajabizadeh M, Rastegar-Pouyani E, Jablonski D, et al. Kukri snakes *Oligodon* Fitzinger, 1826 of the Western Palearctic with the resurrection of Contia transcaspica Nikolsky, 1902 (Reptilia, Squamata, Colubridae). PeerJ. 2023 May 18;11:e15185–5.

29. Leviton AE, Anderson SC. The Herpetological Literature for southwestern Asia. second edition. Vol. 161. USA: California academy of sciences; 2013.

30. Moradi N, Masjedi F, Mahdi R. Study of Snakes species diversity of Sari area, Mazandaran Province. In: 16th National and 4th International Conference of Biology. Mashhad, Iran: Ferdowsi University; 2010.

31. Moradi N, Rastegar-Pouyani N, Rastegar-Pouyani E. Geographic variation in the morphology of *Macrovipera lebetina* (Linnaeus, 1758) (Ophidia: Viperidae) in Iran. Acta herpetologica [Internet]. 2014 Dec 10 [cited 2024 Jul 20];9(2):187–202. Available from: https://doi.org/10.13128/Acta_Herpetol-14384

32. Moradi N, Shafiei Bafti S, Sehhatisabet M, Zadhoush B, Rastegar-Pouyani E. Contribution to the knowledge of the genus *Lytorhynchus* Peters, 1863 (Reptilia: Squamata: Colubridae) with special reference to the Iranian taxa. J Anim Divers [Internet]. 2023 Oct 10;5(4):15–31. Available from: http://jad.lu.ac.ir/article-1-308-en.html

33. Moradi N, Shafiei S, Sehhatisabet M. The snake fauna of Khabr National Park, southeast of Iran. IJAB [Internet]. 2013 Apr 1;9(1):41–55. Available from: http://dx.doi.org/10.22067/ijab.v9i1.33305

34. Moradi SH, Rastegar Pouyani‬ E, Hosseinian Yousefkhani SS, Zargan J. Phenotypic and geographic variation among populations of the *Platyceps rhodorachis-ventromaculatus* species complex (Ophidia: Colubridae) in Iran. IJAB [Internet]. 2022 Dec 1;18(2):171–84. Available from: https://ijab.um.ac.ir/article_43215.html

35. Moradi SH, Rastegar Pouyani E, Hosseinian Yousefkhani SS, Zargan J. Evaluation ecological niche between *Platyceps rhodorachis* and *P. karelini* (Serpentes: Colubridae) in Iran. IJAB [Internet]. 2021 Dec 1;17(2):147–55. Available from: https://ijab.um.ac.ir/article_41624.html

36. Nabavi S, Kami H, Hojati V. The faunestic study of Reptiles in Miankaleh wildlife refuge in Mazandaran Province. J Anim Biol. 2013 Nov;6(1):77–87.

37. Nasrabadi R, Rastegar-Pouyani E, Hosseinian Yousefkhani S, Khani A. Reptiles Fauna of Sabzevar, Northeastern Iran. IJAB [Internet]. 2016 May 1;12(2):255–9. Available from: https://ijab.um.ac.ir/article_28622.html

38. Nilson G, Rastegar-Pouyani N. On the occurrence of *Eirenis coronelloides* (Jan, 1862) in western Iran (Reptilia: Colubridae). Zool Middle East [Internet]. 2011 Jan 1;54(1):133–5. Available from: https://doi.org/10.1080/09397140.2011.10648886

39. Nilson G, Rastegar-Pouyani N. The occurence of *Telescopus nigriceps* (Ahl, 1924) in western Iran, with comments on the genusTelescopus(Serpentes: Colubridae). Zool Middle East [Internet]. 2013 Jun 1;59(2):131–5. Available from: https://doi.org/10.1080/09397140.2013.810874

40. Rajabizadeh M, Kami H. Population study of *Gloydius halys caucasicus* (Nikolsky, 1916) (Reptilia:Viperidae) in central and eastern Elburz, Iran. In: 2nd National Conference of Animal Science. Rasht, Iran: Guilan University; 2007. p. 189–90.

41. Rajabizadeh M, Nagy ZT, Adriaens D, Avci A, Masroor R, Schmidtler J, et al. Alpine-Himalayan orogeny drove correlated morphological, molecular, and ecological diversification in the Persian dwarf snake (Squamata: Serpentes: *Eirenis persicus*). Zool J Linn Soc. 2015 Nov 20;176(4):878–913.

42. Rajabizadeh M, Nilson G, Kami HG, Naderi AR. Distribution of the subgenus *Acridophaga* Reuss, 1927 (Serpentes: Viperidae) in Iran. IJAB [Internet]. 2011 Sep 1;7(1):83–7. Available from: https://ijab.um.ac.ir/article_25536.html

43. Rajabizadeh M, Rastegar-Pouyani N. Additional information on the distribution and morphology of *Coluber* (s. l.) *andreanus* (Werner, 1917) (Reptilia: Colubridae) from Iran. Zool Middle East [Internet]. 2006 Jan;39(1):69–74. Available from: https://doi.org/10.1080/09397140.2006.10638184

44. Rajabizadeh M, Schmidtler JF, Orlov N, Soleimani G. Review of taxonomy and distribution of the *Eirenis medus* group (Chernov, 1940) (Ophidia: Colubridae) with description of a new species of the genus Eirenis from Kerman Province, Southeastern Iran. Russ J Herpetol [Internet]. 2012 Dec 7;19(4):307–13. Available from: https://doi.org/10.30906/1026-2296-2012-19-4-307-313

45. Rajabizadeh M. (2017). Snakes of Iran. Tehran: Iranshenasi. Persian.

46. Ramezani M, Kami H, Ahmadpanah N. Faunistic studies on snakes of West Golestan state. Procedia - Social and Behavioral Sciences [Internet]. 2011;19:811–7. Available from: https://doi.org/10.1016/j.sbspro.2011.05.199

47. Rastegar-Pouyani E, Ebrahimipour F, Hosseinian S. Genetic variability and differentiation among the populations of Dice snake, *Natrix tessellata* (Sepentes, Colubridae) in the Iranian Plateau. Biochem Syst Ecol [Internet]. 2017 Jun;72:23–8. Available from: https://doi.org/10.1016/j.bse.2017.02.014

48. Sadeghi N, Rajabizadeh M, Rastegar-Pouyani N, Hosseinian Yousefkhani S. Update distribution of *Eirenis collaris* (Ménétriés, 1832) (Serpentes; Colubridae) in Iran. Herpetol Notes. 2014 Apr;7:245–6.

49. Safaei-Mahroo B, Gaffari H. New data on presence of the smooth snake *Coronella austriaca* Laurenti, 1768 (Serpentes: Colubridae) in Iran with notes on habitat. Herpetol Notes. 2015 Apr 19;8:235–8.

50. Salemi A, Heydari N, Mahin M. A new distribution record for the rare Maynard’s Longnose Sand Snake, *Lytorhynchus maynardi* Alcock and Finn, 1896 from Nikshahr, southeastern Iran. Herpetol Notes. 2018 Aug 15;11:617–9.

51. Schätti B, Kucharzewski C, Tillack F. *Platyceps rhodorachis* (JAN, 1863) - a study of the racer genus Platyceps BLYTH, 1860 east of the Tigris (Reptilia: Squamata: Colubridae). Vertebr Zool [Internet]. 2014 Dec 5;64(3):297–405. Available from: https://doi.org/10.3897/vz.64.e31498

52. Shafiei Bafti S, Sehhatisabet ME, Moradi N. A range extension of *Walterinnesia morgani* (Mocquard, 1905)(Reptilia: Squamata: Elapidae) to Southeastern Iran. J Anim Divers. 2023 Oct 10;5(3):19-25.

53. Shafiei S., SehatiSabet M.E., Moghadas D. Major Vertebrates Fauna of Bidoieh Protected Area, Kerman Province. J Environ Sci Stud [Internet]. 2004;30(34):71-88. Available from: https://sid.ir/paper/3196/en

54. Sindaco R, Venchi A, Grieco C. The Reptiles of the Western Palearctic: 2. Annotated checklist and distributional atlas of the snakes of Europe, North Africa, Middle East and Central Asia. Latina: Edizioni Belvedere; 2013.

55. Yadollahvandmiandoab R, Farashi A, Ebrahimi A. Species distribution modeling of two poorly known populations of Spalerosophis diadema (Reptilia: Colubridae): are the Zagros Mountains a true barrier for fragmentation in this species? Geol ecol landsc [Internet]. 2023 Sep 4;1–13. Available from: <https://doi.org/10.1080/24749508.2023.2254007>

56. Yousefi M, Khani A, Ilanlo S. Reptiles’ fauna of the Khajeh protected area. In: 16th National and 4th International Conference of Biology. Mashhad, Iran: Ferdowsi University; 2010.

57. Yousefkhani SS, Yousefi M, Khani A, Pouyani ER. Snake fauna of Shirahmad wildlife refuge and Parvand protected area, Khorasan Razavi province, Iran. Hepetol Notes. 2014;7:75-82.

58. Zadhoush B, Van Den Brink M, Rajabizadeh M. Geographic Distribution: *Eirenis rechingeri*. Herpetol Rev. 2016;47(2):262.
