# Supplementary material for: Biogeography of the Iranian snakes
Source: PLoS One. 2024 Oct 16;19(10):e0309120. doi: 10.1371/journal.pone.0309120 (PMC11482698; doi:10.1371/journal.pone.0309120)
Supplement: S2 Table — Climate Types [1], Main Biomes and Ecoregions [2]. (DOCX) [file pone.0309120.s003.docx]

S2 Table.

| **Biogeographical characteristics** | **Physiographic regions** | | | | | | | | | | | | |  |
| --- | --- | --- | --- | --- | --- | --- | --- | --- | --- | --- | --- | --- | --- | --- |
|  | Ce | Ca | A | M | R | Z | WZ | Kh | T | K | S | B | I | |
| **Climate Type** [1] |  |  |  |  |  |  |  |  |  |  |  |  |  | |
| Arid, with cold season | * |  | * | * | * |  |  |  | * | * |  |  |  | |
| Mediterranean, winter rains | * | * | * | * | * | * | * |  | * | * |  |  |  | |
| Subtropical, hot and arid | * |  |  |  |  |  |  | * | * | * | * | * | * | |
| Warm-temperate, humid |  | * |  |  |  |  |  |  | * |  |  |  |  | |
| **Main Biomes** [2] |  |  |  |  |  |  |  |  |  |  |  |  |  | |
| Deserts and Xeric Shrublands | * |  | * | * |  | * | * | * | * | * | * | * | * | |
| Flooded Grassland and Savannas |  |  |  |  |  |  |  | * |  |  |  |  |  | |
| Montane Grasslands and Shrublands | * |  |  | * |  |  |  |  |  | * | * | * |  | |
| Temperate Broadleaf and Mixed Forests |  | * | * | * | * | * | * |  |  |  |  |  |  | |
| Temperate Coniferous Forests |  |  | * | * |  |  |  |  |  | * |  |  |  | |
| Temperate Grasslands, Savannas and Shrublands |  |  | * | * | * |  |  |  |  |  |  |  |  | |
| **Ecoregions** [2] |  |  |  |  |  |  |  |  |  |  |  |  |  | |
| Alborz Range forest steppe |  |  | * | * | * |  |  |  |  | * |  |  |  | |
| Arabian Desert and East Saharo-Arabian xeric shrublands |  |  |  |  |  |  |  | * |  |  |  |  |  | |
| Azerbaijan shrub desert and steppe |  |  |  | * |  |  |  |  |  |  |  |  |  | |
| Badkhiz-Karabil semi-desert |  |  |  |  |  |  |  |  |  | * |  |  |  | |
| Caspian Hyrcanian mixed forests |  | * | * | * |  |  |  |  |  |  |  |  |  | |
| Caspian lowland desert |  |  |  |  |  |  |  |  | * |  |  |  |  | |
| Central Persian desert basins | * |  | * |  |  | * |  |  |  | * |  |  |  | |
| Eastern Anatolian montane steppe |  |  | * | * | * |  |  |  |  |  |  |  |  | |
| Kopet Dagh semi-desert |  |  |  |  |  |  |  |  | * |  |  |  |  | |
| Kopet Dagh woodlands and forest steppe |  |  |  |  |  |  |  |  |  | * |  |  |  | |
| Kuh Rud and Eastern Iran Montane woodlands | * |  |  |  |  | * |  |  |  | * |  | * |  | |
| Mesopotamian shrub desert |  |  |  |  |  |  | * |  |  |  |  |  |  | |
| Registan-North Pakistan sandy desert |  |  |  |  |  |  |  |  |  |  | * | * |  | |
| South Iran Nubo-Sindian desert and semi-desert |  |  |  |  |  | * | * | * |  |  |  | * | * | |
| Tigris-Euphrates alluvial salt marsh |  |  |  |  |  |  |  | * |  |  |  |  |  | |
| Zagros Mountains forest steppe |  |  | * |  | * | * | * |  |  |  |  |  |  | |

References

1. Walter, H., Lieth, H. Klimadiagramm-Weltatlas. Jena: Fischer-Verlag; 1960–1967.

2. Olson DM, Dinerstein E, Wikramanayake ED, Burgess ND, Powell GVN, Underwood EC, et al. Terrestrial Ecoregions of the World: A New Map of Life on Earth. BioScience [Internet]. 2001;51(11):933. Available from: <https://academic.oup.com/bioscience/article/51/11/933/227116>
