# Supplementary material for: Biogeography of the Iranian snakes
Source: PLoS One. 2024 Oct 16;19(10):e0309120. doi: 10.1371/journal.pone.0309120 (PMC11482698; doi:10.1371/journal.pone.0309120)
Supplement: S3 Table — The data are clustered using UPGMA and presented as dendrogram in Fig 4A. (DOCX) [file pone.0309120.s004.docx]

S3 Table.

|  | Sa | O | W | Tu | Ar | Ir | Ce | Ca | A | M | R | Z | WZ | Kh | T | K | S | B |
| --- | --- | --- | --- | --- | --- | --- | --- | --- | --- | --- | --- | --- | --- | --- | --- | --- | --- | --- |
| O | 0.7 |  |  |  |  |  |  |  |  |  |  |  |  |  |  |  |  |  |
| W | 0.536 | 0.679 |  |  |  |  |  |  |  |  |  |  |  |  |  |  |  |  |
| Tu | 0.565 | 0.55 | 0.37 |  |  |  |  |  |  |  |  |  |  |  |  |  |  |  |
| Ar | 0.368 | 0.773 | 0.552 | 0.64 |  |  |  |  |  |  |  |  |  |  |  |  |  |  |
| Ir | 0.542 | 0.591 | 0.467 | 0.48 | 0.5 |  |  |  |  |  |  |  |  |  |  |  |  |  |
| Ce | 0.474 | 0.684 | 0.462 | 0.476 | 0.421 | 0.3 |  |  |  |  |  |  |  |  |  |  |  |  |
| Ca | 0.75 | 0.895 | 0.615 | 0.667 | 0.818 | 0.8 | 0.8 |  |  |  |  |  |  |  |  |  |  |  |
| A | 0.56 | 0.815 | 0.192 | 0.5 | 0.577 | 0.586 | 0.6 | 0.524 |  |  |  |  |  |  |  |  |  |  |
| M | 0.708 | 0.875 | 0.385 | 0.583 | 0.72 | 0.714 | 0.696 | 0.375 | 0.238 |  |  |  |  |  |  |  |  |  |
| R | 0.696 | 0.917 | 0.423 | 0.68 | 0.708 | 0.704 | 0.682 | 0.529 | 0.286 | 0.176 |  |  |  |  |  |  |  |  |
| Z | 0.478 | 0.808 | 0.296 | 0.593 | 0.435 | 0.4 | 0.455 | 0.696 | 0.292 | 0.5 | 0.409 |  |  |  |  |  |  |  |
| WZ | 0.45 | 0.826 | 0.385 | 0.583 | 0.4 | 0.435 | 0.421 | 0.7 | 0.318 | 0.545 | 0.45 | 0.2 |  |  |  |  |  |  |
| Kh | 0.364 | 0.769 | 0.433 | 0.607 | 0.238 | 0.423 | 0.478 | 0.76 | 0.444 | 0.63 | 0.56 | 0.292 | 0.238 |  |  |  |  |  |
| T | 0.609 | 0.6 | 0.407 | 0.25 | 0.68 | 0.52 | 0.524 | 0.579 | 0.48 | 0.565 | 0.609 | 0.577 | 0.565 | 0.643 |  |  |  |  |
| K | 0.5 | 0.667 | 0.259 | 0.304 | 0.52 | 0.36 | 0.333 | 0.652 | 0.444 | 0.52 | 0.56 | 0.36 | 0.391 | 0.444 | 0.348 |  |  |  |
| S | 0.55 | 0.611 | 0.571 | 0.609 | 0.421 | 0.3 | 0.353 | 0.857 | 0.654 | 0.8 | 0.792 | 0.522 | 0.5 | 0.542 | 0.652 | 0.478 |  |  |
| B | 0.55 | 0.529 | 0.571 | 0.609 | 0.421 | 0.3 | 0.353 | 0.857 | 0.654 | 0.8 | 0.792 | 0.522 | 0.5 | 0.478 | 0.652 | 0.478 | 0.133 |  |
| I | 0.647 | 0.882 | 0.786 | 0.818 | 0.5 | 0.727 | 0.625 | 0.875 | 0.792 | 0.857 | 0.85 | 0.727 | 0.737 | 0.619 | 0.864 | 0.739 | 0.625 | 0.625 |
