# Supplementary material for: Biogeography of the Iranian snakes
Source: PLoS One. 2024 Oct 16;19(10):e0309120. doi: 10.1371/journal.pone.0309120 (PMC11482698; doi:10.1371/journal.pone.0309120)
Supplement: S4 Table — The data are clustered using UPGMA and presented as dendrogram in Fig 4B. (DOCX) [file pone.0309120.s005.docx]

S4 Table

|  | Sa | O | W | Tu | Ar | Ir | Ce | Ca | A | M | R | Z | WZ | Kh | T | K | S | B |
| --- | --- | --- | --- | --- | --- | --- | --- | --- | --- | --- | --- | --- | --- | --- | --- | --- | --- | --- |
| O | 1 |  |  |  |  |  |  |  |  |  |  |  |  |  |  |  |  |  |
| W | 0.932 | 0.986 |  |  |  |  |  |  |  |  |  |  |  |  |  |  |  |  |
| Tu | 1 | 0.963 | 0.708 |  |  |  |  |  |  |  |  |  |  |  |  |  |  |  |
| Ar | 0.727 | 0.958 | 0.861 | 0.868 |  |  |  |  |  |  |  |  |  |  |  |  |  |  |
| Ir | 0.951 | 0.857 | 0.75 | 0.739 | 0.804 |  |  |  |  |  |  |  |  |  |  |  |  |  |
| Ce | 0.926 | 0.96 | 0.77 | 0.667 | 0.758 | 0.444 |  |  |  |  |  |  |  |  |  |  |  |  |
| Ca | 1 | 1 | 0.757 | 0.71 | 0.943 | 0.96 | 0.944 |  |  |  |  |  |  |  |  |  |  |  |
| A | 0.911 | 1 | 0.458 | 0.638 | 0.87 | 0.754 | 0.708 | 0.585 |  |  |  |  |  |  |  |  |  |  |
| M | 0.933 | 1 | 0.657 | 0.795 | 0.952 | 0.927 | 0.875 | 0.481 | 0.523 |  |  |  |  |  |  |  |  |  |
| R | 0.933 | 1 | 0.657 | 0.825 | 0.952 | 0.927 | 0.875 | 0.633 | 0.587 | 0.345 |  |  |  |  |  |  |  |  |
| Z | 0.902 | 0.961 | 0.427 | 0.817 | 0.786 | 0.617 | 0.698 | 0.86 | 0.587 | 0.759 | 0.714 |  |  |  |  |  |  |  |
| WZ | 0.784 | 1 | 0.592 | 0.846 | 0.61 | 0.8 | 0.792 | 0.851 | 0.7 | 0.78 | 0.729 | 0.482 |  |  |  |  |  |  |
| Kh | 0.758 | 0.914 | 0.744 | 0.88 | 0.394 | 0.667 | 0.714 | 0.936 | 0.806 | 0.904 | 0.882 | 0.603 | 0.478 |  |  |  |  |  |
| T | 0.968 | 0.964 | 0.694 | 0.192 | 0.842 | 0.745 | 0.676 | 0.633 | 0.617 | 0.737 | 0.8 | 0.8 | 0.827 | 0.86 |  |  |  |  |
| K | 0.943 | 0.938 | 0.68 | 0.375 | 0.805 | 0.578 | 0.485 | 0.821 | 0.6 | 0.822 | 0.822 | 0.695 | 0.778 | 0.784 | 0.394 |  |  |  |
| S | 0.963 | 0.864 | 0.861 | 0.735 | 0.788 | 0.429 | 0.536 | 0.972 | 0.827 | 0.977 | 0.977 | 0.786 | 0.882 | 0.738 | 0.706 | 0.6 |  |  |
| B | 0.926 | 0.818 | 0.863 | 0.743 | 0.758 | 0.4 | 0.552 | 0.973 | 0.808 | 0.977 | 0.977 | 0.789 | 0.863 | 0.714 | 0.714 | 0.611 | 0.217 |  |
| I | 0.714 | 0.929 | 0.947 | 0.969 | 0.696 | 0.929 | 0.893 | 1 | 0.959 | 1 | 1 | 0.885 | 0.795 | 0.697 | 0.97 | 0.917 | 0.889 | 0.893 |
